# Genome-Wide Comparison Reveals a Probiotic Strain *Lactococcus lactis* WFLU12 Isolated from the Gastrointestinal Tract of Olive Flounder (*Paralichthys olivaceus*) Harboring Genes Supporting Probiotic Action

**DOI:** 10.3390/md16050140

**Published:** 2018-04-24

**Authors:** Thanh Luan Nguyen, Do-Hyung Kim

**Affiliations:** 1 Department of Aquatic Life Medicine, College of Fisheries Science, Pukyong National University, Busan 48513, Korea; nt.luan@hutech.edu.vn; 2 Institute of Applied Science, Ho Chi Minh City University of Technology, Ho Chi Minh City 700000, Vietnam

**Keywords:** probiotics, genes, pan-genome analysis, comparative genomics, carbohydrate utilization

## Abstract

Our previous study has shown that dietary supplementation with *Lactococcus lactis* WFLU12 can enhance the growth of olive flounder and its resistance against streptococcal infection. The objective of the present study was to use comparative genomics tools to investigate genomic characteristics of strain WFLU12 and the presence of genes supporting its probiotic action using sequenced genomes of *L. lactis* strains. Dispensable and singleton genes of strain WFLU12 were found to be more enriched in genes associated with metabolism (e.g., energy production and conversion, and carbohydrate transport and metabolism) than pooled dispensable and singleton genes in other *L. lactis* strains, reflecting WFLU12 strain-specific ecosystem origin and its ability to metabolize different energy sources. Strain WFLU12 produced antimicrobial compounds that could inhibit several bacterial fish pathogens. It possessed the nisin gene cluster (nisZBTCIPRKFEG) and genes encoding lysozyme and colicin V. However, only three other strains (CV56, IO-1, and SO) harbor a complete nisin gene cluster. We also found that *L. lactis* WFLU12 possessed many other important functional genes involved in stress responses to the gastrointestinal tract environment, dietary energy extraction, and metabolism to support the probiotic action of this strain found in our previous study. This strongly indicates that not all *L. lactis* strains can be used as probiotics. This study highlights comparative genomics approaches as very useful and powerful tools to select probiotic candidates and predict their probiotic effects.

## 1. Introduction

Colonization of vast and various communities of microbes is necessary for nutrition uptake and well-being of fish. The intestine of fish is rich in nutrients. It provides a favorable growth environment for numerous adapted bacteria that form unique characteristics of the fish intestine [1,2]. Although lactic acid bacteria (LAB) are not predominantly present among normal microbiota in the gastrointestinal tract of fish [3], numerous LABs have been isolated from the wild population of olive flounder in our previous study [4], indicating that these species might have been highly adapted to the gastrointestinal tract of fish. They might help the host maintain homeostasis of its intestinal ecosystem [5]. Among these LABs, *Lactococcus lactis* WFLU12 can confer farmed olive flounder protection against streptococcal infections. It can also improve fish growth by enhancing some enzyme activities (including phosphohydrolase and glycosidase) in the fish gut [6].

Complete genome sequences of dairy- and nondairy-*L. lactis* strains have been published in the last decade. They might provide some important traits for various applications. For example, they have contributed to the development of fermented dairy products and potential delivery vectors for various antigens with pharmaceutical applications [7]. Numerous studies have demonstrated that *L. lactis* strains exhibit genome-scale diversity with a gene pool that plays a pivotal role in evolution and environmental adaptation [8,9,10]. In a previous study [11], 36 strains of *L. lactis* isolated from different ecological sources and geographical areas showed high variability at both gene and genome levels, prompting a new classification based on ecological separation of “domesticated” and “environmental” lactococcal strains. Genome comparison between *L. lactis* strains isolated from dairy and plant sources has revealed that they have very flexible genomes, showing variability in terms of encoded functions of proteolysis, lactose fermentation, citrate uptake, metal ion resistance, and exopolysaccharides biosynthesis [10]. Although it has been demonstrated that some bacterial species isolated from fish possess beneficial effects [12,13], genomic characteristics of fish gut-derived strains are currently unclear. Therefore, the objective of this study was to determine genomic characteristics of strain WFLU12 and compare its genome with sequenced genomes of other *L. lactis* strains to explore niche-specific variations and genes in the gene repertoire of strain WFLU12 that might support its probiotic action.

## 2. Results and Discussion

### 2.1. General Genome Features

The whole genome sequence of strain WFLU12 was obtained using the SMRT sequencing approach. Primary genome assembly was performed using ABySS version 1.5.1. Scaffolding of the primary assembly was performed using SSPACE-LongRead scaffolder version 1.0 [14]. Results revealed that its genome consisted of three scaffolds with an average genome length of ~2.5 Mbp, G+C content of 35.07%, and N50 size of 2,272,213 bp (~2.27 Mb). Analysis using GapFiller version 1.10 [15] revealed no gap in these assembled scaffolds. Genome annotation using the Prokka system revealed that this genome had 2480 protein coding sequences (CDS), 68 tRNA-encoding genes, and 19 rRNA-encoding genes. A list of CDS in *L lactis* WFLU12 annotated using Prokka is shown in Table S1. No plasmid was identified (Table S2). Among these predicted CDS, 1748 (70.5%) can be assigned to subsystems features predicted with the RAST server [16], including those associated with carbohydrate metabolism (340 genes), amino acids and derivatives (256 genes), protein metabolism (176 genes), and cofactors/vitamins/prosthetic groups/pigments (127 genes).

To analyze molecular differences between fish gut isolate and isolates from terrestrial sources, in silico comparative analysis was performed to identify the gene composition and potential probiotic function of strain WFLU12. Based on ANI analysis, strain WFLU12 was identified as *L. lactis* subsp. *lactis* (>98% sequence identities, Table S2) when its genome was compared to all available complete genomes of *Lactococcus* species. Chromosome sequences of 20 strains of subspecies *lactis* (Table S2) were also compared to assess chromosomal diversity.

**Figure 1:**
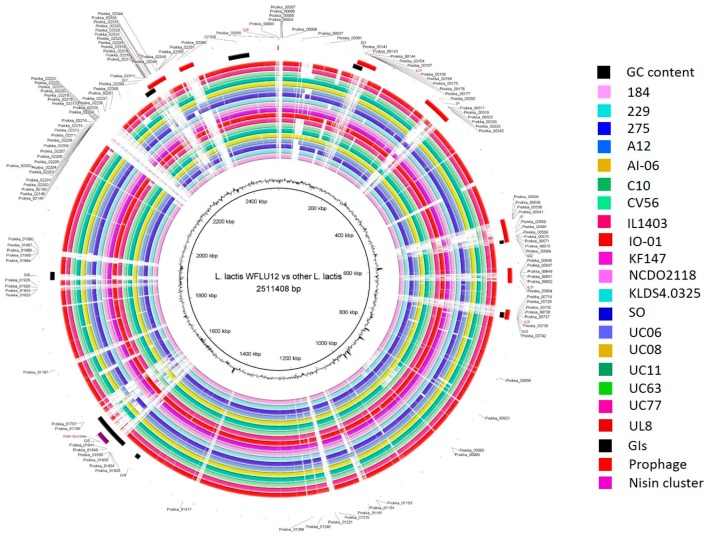
Blast Ring Image Generator (BRIG) diagram showing homologous chromosome segments of *L. lactis* subsp. *lactis* strains with genome of strain WFLU12 as reference. The outer circle contains genomic island regions (black), prophage (red), and nisin cluster (purple) of strain WFLU12. GC content is also shown in the figure. The indicated locus tag represents a stretch of singleton genes in WFLU12 compared to others.

A total of 20 subsp. *lactis* strains used in this study were originally isolated from seven different ecological niches (dairy, plant, meat, fermented foods, human isolate, sink drain, and marine fish gut), suggesting that each strain might have adapted to its ecological niche. Of 2480 protein CDS in the genome of WFLU12, 75.56% were functionally assigned based on Basic Local Alignment Search Tool (BLAST) while the remaining 24.44% were hypothetical proteins. Our results are consistent with results of general genome analysis of subsp. *Lactis* [7], showing that the average number of predicted CDSs is 2344 (range 1947–2643) per chromosome. In addition, 78.4% and 21.6% of CDS are assigned as functional and hypothetical proteins, respectively. Results of ortholog analyses using five selected strains (IL1403, KF147, SO, UC11, and CV56) showed that they had 1870, 1986, 1927, 1948, and 1915 orthologs, respectively. Most WFLU12 genes were also shared by these strains. However, there are also major differences between WFLU12 and others (white gaps in Figure 1). These gaps might be due to integration of mobile elements. Indeed, we were able to identify prophages and genomic islands located in these gaps. This may indicate adaptability to variable environments of low IS harboring strains compared to trends in genome decay and redundancy of high IS harboring strains (mainly from dairy isolates) as suggested by previous studies [17,18,19]. It has been suggested that dairy strains are under industrial pressures of repeated passaging in the nutrient-rich growth medium of milk [19].

**Figure 2:**
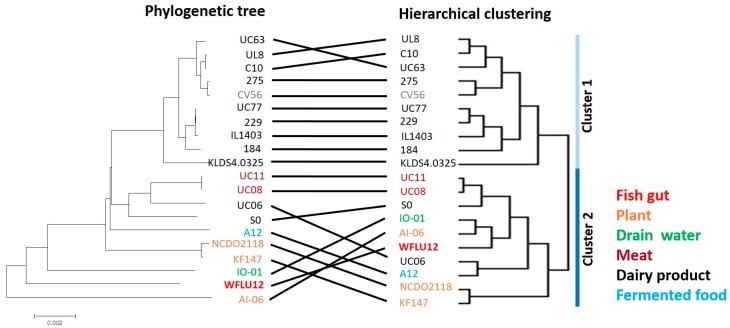
Comparison between hierarchical clustering and phylogenetic tree of *L. lactis* subsp. *lactis* strains. Both hierarchical clustering (right panel) based on shared gene content and phylogenetic tree (left panel) based on concatenated orthologous genes were performed for all 20 strains. Strings connecting the same strains of both trees are used to highlight the degree of similarities between both tree methods [21,22].

Unlike the phylogenetic tree that reflects different evolution within the genome (i.e., point mutations), the use of the hierarchical clustering tree could provide an overview of intra-species phenotypic variation by measuring major genetic re-arrangement events (i.e., insertions and deletions occurring in their natural habitats) [20]. Based on grouping with these two genetic algorithms, we could depict some degree of conservation in the grouping of subspecies *lactis* strains (Figure 2). Remarkably, most dairy strains were found to be well grouped by both genetic algorithms. They belonged to cluster 1 in hierarchical clustering, showing marked differences from the other cluster and high conservation. Remaining strains derived from various sources (except SO and UC06) were also well grouped into cluster 2. A previous study [10] has demonstrated that *L. lactis* strains are diversified depending on isolation sources (dairy and plant) by analyzing the presence/absence of gene clusters related to growth on different carbohydrate substrates, biosynthesis of exopolysaccharides, stress responses (e.g., iron transport, osmotolerance), and bacterial defense mechanisms (nisin biosynthesis). Strain WFLU12 isolated from fish gut shared similarities with drain water (IO-01) and plant isolates (AI-06) of *L. lactis* based on both hierarchical clustering and phylogeny.

### 2.2. Pan-Genomic Analysis of L. lactis Subsp. lactis

**Figure 3:**
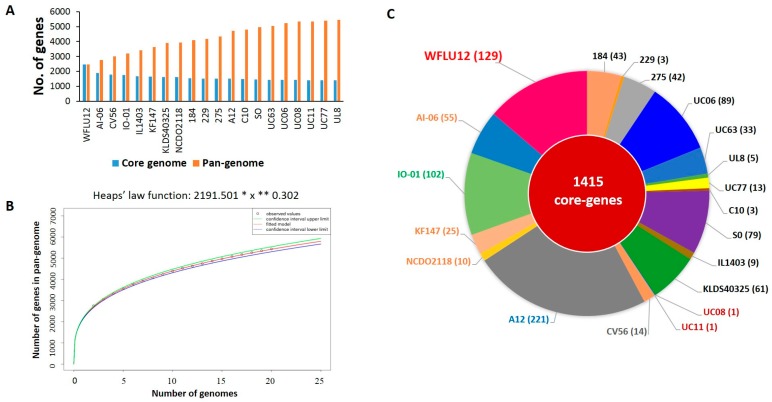
Pan-genome analyses of 20 *L. lactis* subsp. *lactis* strains. (**A**) Sizes of pan-genome and core genome of *L. lactis* subsp. *lactis*. (**B**) Pan-genome development extrapolation of 20 *L. lactis* subsp. *lactis* indicated an open pan-genome according to Heap’s Law model. (**C**) Calculated singleton gene sets to each chromosome. These were extracted using EDGAR [22]. Each strain is labeled by color-coding as indicated in Figure 2.

As shown in the pan-genome graph (Figure 3A), the size of pan-genome inferred from genome sequences of 20 subsp. *lactis* strains was constituted by 5442 orthologous groups. According to Tettelin et al. [23], Heap’s Law function can be used to calculate whether the pan-genome is open or closed. It is represented by the following formula: *n* = k × N^−α^. The power law coefficient within this equation is 0.302 (between 0 and 1), corresponding to the open pan-genome model [23] (Figure 3B). In contrast to a previous study [7] showing that 30 lactococcal strains (including subsp. *lactis* and *cremoris*) were in a closed state, an open pan-genome model was applicable to subsp. *lactis* in this study. Thus, an addition of our sequenced genome will contribute to the characterization of a new gene repertoire of this subspecies.

Based on such pan-genome distribution, we divided all orthologous groups into three groups: core (present in all 20 subsp. *lactis* strains), dispensable (present in at least two strains, but not all), and singleton genes (seen in only one of 20 strains). Consistent with 1413 core-genes outlined in a previous study for 18 strains of subsp. *lactis* [7], the core genome contained 1415 genes in a total of 20 genomes used in this study (Figure 3C). This indicated that two additional genomes (strains WFLU12 and A12) might have increased dispensable and singleton genes in their open pan-genome size. A total of 938 singleton genes were calculated across these 20 subsp. *lactis* strains assessed. Strain A12 harbored the highest number of singleton genes (221 genes), followed by strain WFLU12 with 129 genes. The genome of WFLU12 presented a longer stretch of singleton genes in the white gapped blast area compared to the other 19 strains (Figure 1). Predicted genomic islands and prophage-like elements search in WFLU12 genome were located in positions of singleton genes, suggesting that these singleton genes might have been foreign in nature. They might have been inherited by horizontal gene transfer events.

### 2.3. Functional Comparative Analysis of Pan- and Core-Genomes

**Figure 4:**
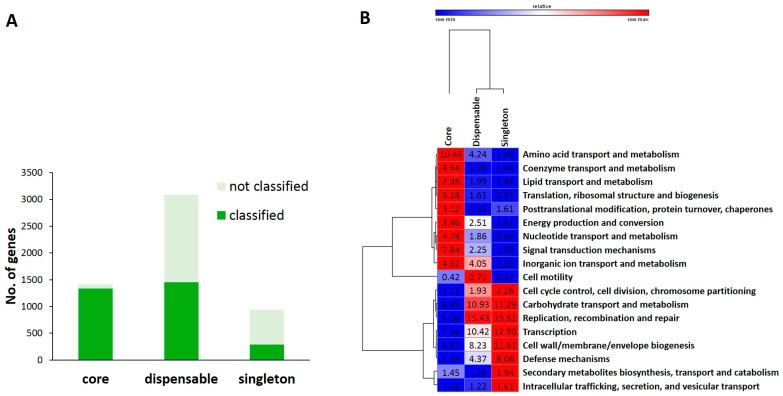
Functional annotation of ortholog groups in different parts of the pan-genome of *L. lactis* subsp. *lactis*. (**A**) Distribution of ortholog groups (functionally annotated or not) in different parts of the pan-genome using a search in the COG database. (**B**) Scaled heat map showing the distribution of functional classes among different parts of the pan-genome.

To determine functional differences between genomes, we performed re-annotation of all genomic subsets using PSI-BLAST against NCBI COG database (Figure 4). We obtained 1337, 1452, and 289 clusters with predicted functions for core, dispensable, and singleton genes, respectively. The rest (43%) of orthologous groups could not be annotated using the COG database. Most of these genes were located among dispensable and singleton genes (Figure 4A). Figure 4B shows non-uniform distribution of many predicted functional classes among functionally annotated genes. The core genome was found to be overrepresented by genes involved in the following functional classes: (1) translation, (2) ribosomal structure and biogenesis, and (3) amino acid transport and metabolism. In contrast, dispensable genes were overrepresented by genes belonged to functional classes such as replication/recombination/repair, carbohydrate transport and metabolism, transcription, and cell envelope biogenesis. Genes involved in replication, transcription, cell envelope biogenesis, carbohydrate transport, and metabolism were enriched in pooled singleton genes. This indicates that genes related to information storage processes (replication, recombination, and repair, and transcription) are more variable in the pan-genome but less variable in the core genome than genes related to translation and ribosome biogenesis. For functional groups of mobile elements such as transposase and site-specific recombinases, distribution of these genes was found to be nearly strain-specific. In addition, gene content for metabolism of amino acids was more conserved than that for carbohydrates. High frequency of genes for cell envelope biogenesis reflected high levels of various glycosyltransferases, carbohydrate modification proteins, and sortases. Carbohydrate transport and metabolism genes were also more frequently found in dispensable and singleton genes than those in core genes. This is also observed in other species such as *Bifidobacterium* and *Lactobacillus* [24,25].

From pan-genome analysis of strain WFLU12 (Figure S1), we obtained 536 clusters with predicted functions from 936 dispensable genes and 49 clusters from 129 singleton genes. The rest (22.5%) belonged to unclassified genes from dispensable and singleton genes. Similar to results of the pooled pan genome, dispensable and singleton genes of strain WFLU12 consisted of several functional classes belonging to transcription or carbohydrate transport and metabolism with higher frequencies. We also found that both dispensable and singleton genes of strain WFLU12 harbored more genes related to metabolism (energy production and conversion, carbohydrate transport and metabolism) but less genes involved in information storage (replication, recombination, and repair) than those of pooled dispensable and singleton genes of *L. lactis* strains. Differences between these genes involved in metabolism could indicate a signature of strain-specific ecosystem origin and its ability to metabolize different energy sources. Groups of metabolism related genes (including those associated with amino acid transport and metabolism, coenzyme transport and metabolism, and inorganic ion transport and metabolism) were more frequently found in dispensable genes of strain WFLU12 than those in pooled dispensable genes. Lastly, enrichment in singleton genes related to cell wall/membrane/envelope biogenesis in strain WFLU12 was apparent within the group of ‘cellular processes and signaling’. This might indicate the importance of such genes in the natural habitat of gut bacteria.

### 2.4. Comparing Abundance of Cazy among Strains

**Figure 5:**
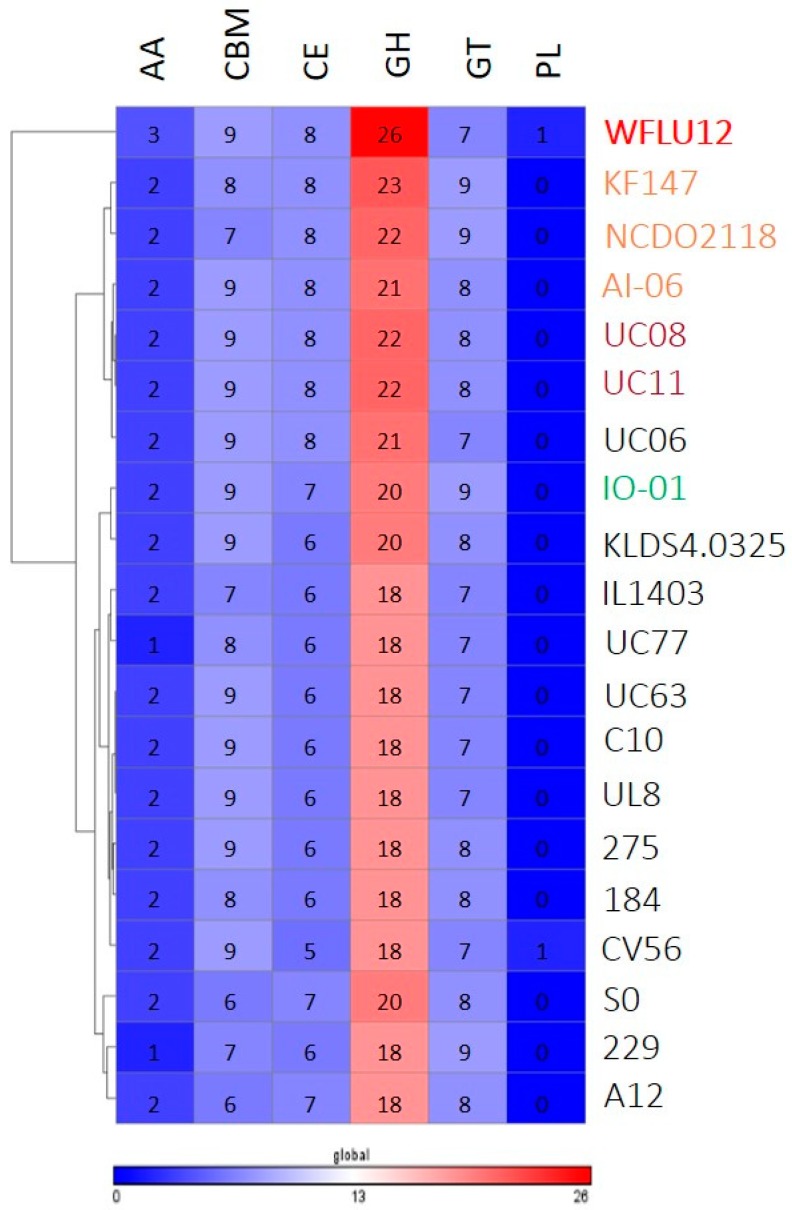
Comparison of carbohydrate-active enzyme (CAZyme) families between genome of strain WFLU12 and 19 other *L. lactis* genomes. Numbers of enzyme modules in each genome are shown. Over-represented (red) and under-represented modules (blue) are depicted based on the number of repertoires. Each strain is labeled by color-coding as indicated in Figure 2. Abbreviated name of CAZy families: GH, Glycoside Hydrolases; GT, Glycosyl Transferases; PL, Polysaccharide Lyases; CE, Carbohydrate Esterases; CBM, Carbohydrate Binding Modules; AA, Auxiliary Activity.

Generally, the range of polysaccharide substrates that arrives in the intestine from the diet is enormous [26]. Gut bacteria with a broad capacity to metabolize complex sugars have selective advantage that allows them to fill their ecological niche. Genome annotation confirmed that *L. lactis* had a variety of genes. Their gene clusters are specialized in fermentation of a wide spectrum of complex sugars (e.g., plant polysaccharides materials, like cellulose, xylan, pectin, and arabinan) other than lactose as growth substrates in a dairy environment [27,28,29], suggesting growth adaptation of *L. lactis* to variable substrates derived from their natural niches. In this study, a total of 117 CAZy were identified in the genome of *L. lactis* WFLU12. This number is similar to the number of CAZy found in other strains, ranging from 94 to 126 (Table S3). In particular, this analysis showed that strain WFLU12 had some CAZy families not found in other strains, such as AA6, CMB12, GH105, GH24, GH28, GH35, GH88, and PL8 (Table S3), indicating a distinctive CAZy profile of strain WFLU12 (Figure 5). In line with previous studies [10,27,29], these novel CAZy families/genes in strain WFLU12 belonged to a singleton genes repertoire comprising most strain-specific genes involved in carbohydrate metabolism. They are potentially required for its persistence in different ecological niches.

### 2.5. Specificity in the Genome of Strain WFLU12

Unique gene and gene clusters in strain WFLU12 included sugar metabolism (i.e., gluconate 5-dehydrogenase, polygalacturonase, rhamnulokinase), cell envelope biogenesis (i.e., fimbrial subunit type 2, teichoic acid biosynthesis), antagonistic activity (i.e., lantibiotic transport, lysozyme), and many hypothetical proteins (Table S4). The genome of WFLU12 harbors a cluster of genes encoding several enzymes (e.g., l-rhamnose isomerase (RhaA, EC 5.3.1.14); l-rhamnulose kinase (RhaB, EC 2.7.1.5); and l-rhamnulose-1-phosphate aldolase (RhaD, EC 4.1.2.19)) involved in the metabolism of l-rhamnose [30,31,32] (CYU10_0CYU10_002215–02225) (Table S4). To the best of our knowledge, this is the first study showing that the *L. lactis* strain possesses a gene cluster encoding enzymes for l-rhamnose metabolism. In this study, strain WFLU12 was grown on a medium containing l-rhamnose as shown in Table S5. Previous studies [32,33] have demonstrated that l-rhamnose isomerase has very broad specificity toward various substrates, including l-lyxose, aldoses, and ketoses. This indicates that strain WFLU12 is likely to degrade these rare carbohydrates.

Non-uniform CAZy profiles analysis showed that *L. lactis* strains were well clustered depending on isolation source. Our strain WFLU12 could be distinguished from other strains (Figure S2). Of 20 glycoside hydrolase (GH) families, five GH families were only present in WFLU12. These five families included families GH24, GH28, GH88, GH105, and GH35 representing lysozyme, polygalacturonase, unsaturated chondroitin disaccharide hydrolase, unsaturated rhamnogalacturonyl hydrolase, and beta-galactosidase-1-like protein 2 acting on different galactose-containing molecules, respectively. Most strains (except WFLU12 and IO-01) harbored β-galactosidases that belonged to family GH2. However, only strain WFLU12 harbored a gene encoding enzyme for GH35 β-galactosidases, indicating its potential to act on different galactose-containing glycosides substrates. Indeed, GH35 β-galactosidases can act on higher oligosaccharide and polysaccharides such as polymers of galactose (galactans) [34,35]. The marine environment contains a wide range of complex polysaccharides, including galactans and rhamnose [36]. Galactans are major polysaccharides as constituents of many seaweed species. Rhamnose is one of major constituents of rhamnogalacturonans found in pectin-like complex polysaccharide of marine materials (e.g., green algae) [36,37]. Unique genes encoding enzymes for l-rhamnose metabolism and GH35 β-galactosidases were only found in our strain WFLU12, indicating niche-specific adaptation of strain WFLU12 to marine environment. These unique characteristics might give it an advantage over other *L. lactis* strains to use a variety of carbohydrate complexes in its environment.

### 2.6. Genes Potentially Involved in Probiotic Effects of Strain WFLU12

It has been shown that dietary supplementation of *L. lactis* can improve disease resistance, growth performance, and feed efficiency in fish [6,38,39]. Strain WFLU12 was regarded as harmless to olive flounder as clinical signs of disease or mortality were not observed in fish challenged with this bacterium through intramuscular or intraperitoneal injection with a long-term monitoring in our previous study [6]. In the era of the genome, it is possible to discover genes responsible for probiotic activities such as safety aspects, ability to colonize and survive in the intestinal ecological niche, and ability to outperform potential pathogenic microbes.

Pathogenicity and antibiotic resistance islands were not present in strain WFLU12, corroborating its safety aspects. This strain was susceptible to most antibiotics assayed in this study (except sulfamethoxazole/trimethoprim and cephalexin, Table S6), consistent with the presence of antibiotic resistance related genes in this strain (Table S7). Indeed, we found gene encoding beta-lactamase class C (*ampC*) [40] and genes involved in antifolate resistance in this strain. They might confer resistance to cephalexin and trimethoprim, respectively. However, the molecular mechanism of antimicrobial resistance of *L. lactis* to sulfamethoxazole remains unclear [41]. In this study, we observed that most genes related to drug resistance of *L. lactis* belonged to the core genome. It is known that *L. lactis* WFLU12 possesses antimicrobial activity against several fish pathogens [6]. This strain also produces antimicrobial compounds due to the nisin gene cluster (nisZBTCIPRKFEG) and genes encoding lysozyme and colicin V (Table S8). In the present study, only four strains (CV56, IO-1, SO, and WFLU12) possessed a complete nisin gene cluster. Antagonistic activity of nisin has been demonstrated in previous studies [6,42,43,44]. In agreement with a previous report [43], chromosomes of some strains had an incomplete nisin gene cluster due to internal deletion (see purple remark in Figure 1 and Table S8). Thus, these strains cannot produce nisin. Notably, strain WFLU12 possessed genes encoding lysozyme, a hydrolase of peptidoglycan (Table S4), as a singleton gene. WFLU12 also harbored a singleton gene encoding a lantibiotic transport ATP-binding protein. It might improve the movement of bacteriocin out of the cell or into the cell. Consequently, not all *L. lactis* strains had antibacterial activity. This property is clearly advantageous for WFLU12 to compete against other microorganisms in the same habitat.

**Figure 6:**
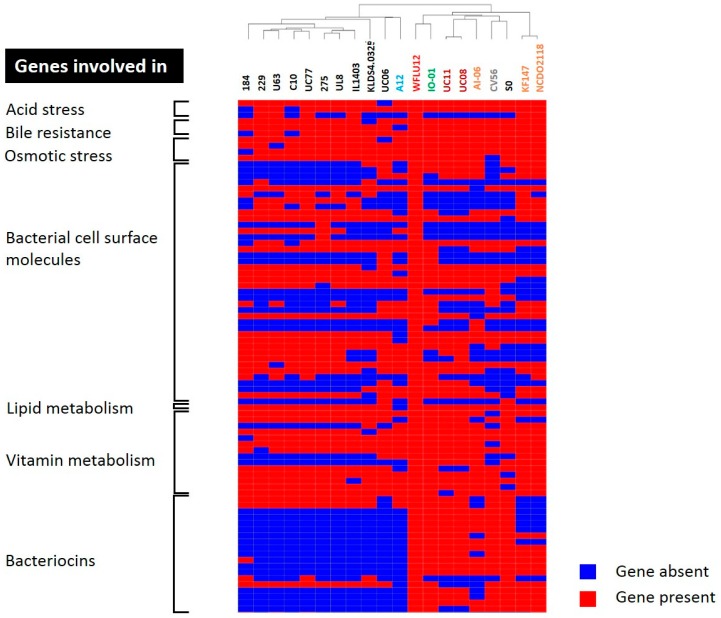
Hierarchical clustering of dispensable genes potentially involved in probiotic effects. The functions of genes were detailed in Tables S8–S10. Presence and absence of genes in each genome are indicated in red and blue, respectively. Each strain is color-coded, similar to those shown in Figure 2.

Probiotic bacteria should have the ability to resist and survive in the gastrointestinal tract which is a stressful condition. Many studies (e.g., [45,46,47,48,49,50]) have already shown that probiotic strains usually harbor genes to resist stressful host environments. Stress responses of probiotic cells rely on coordinated expression or suppression of genes that act in concert to improve stress tolerance. These genes can alter cellular processes such as cell division, membrane composition, transport systems, housekeeping, and DNA metabolism. They are regulated by factors that can control several genes. Sometimes they are regulated by other regulators. In this study, genes of WFLU12 coding for proteins involved in environmental stress were predominantly classified as core genes (55/65 genes, 84.6%) and dispensable genes (10/65 genes, 15.4%) (Table S9), indicating that some *L. lactis* strains might have similar abilities to survive passage through the gastrointestinal tract. Differences in these dispensable genes are shown in hierarchical clustering of gene presence/absence between *L. lactis* strains (Figure 6). This also supports our previous findings that this WFLU12 strain possesses better ability to tolerate acidic conditions (e.g., at pH 2) and high bile salt concentration than *L. lactis* strains isolated from other sources [6].

Genes coding for adhesion-related proteins have been found in *L. lactis* as described previously [51,52]. Seventy-one genes coding for proteins potentially functioning as bacterial cell surface molecules were found in WFLU12 (Table S10). These genes can mediate adhesion to the mucosa of the gastrointestinal tract. Most genes found in this category belonged to dispensable genes (49/71 genes, 69%). Fewer genes in this category were core genes (15/71 genes, 21.1%) or singleton genes (9.9%) (Table S10). These results are consistent with the high frequency of genes involved in cell envelope biogenesis for dispensable and singleton genes as mentioned in Section 2.3 and Figure 4. Cell surface factors confer strain specificity in terms of capacity for adhesive/mucoadhesive behavior [52,53]. In the present study, in silico analysis of adhesion in the WFLU12 genome resulted in two types of surface determinants: pili (CYU10_0CYU10_001788_01802) and mucus-binding proteins (CYU10_0CYU10_002196 and CYU10_0CYU10_002197, Table S10). It has been demonstrated that pili and mucus-binding proteins play an important role in bacterial adhesion to model mucins. Mucus-binding proteins also greatly contribute to bacterial cell-adhesion under shear flow conditions [51,52,54]. Strain WFLU12 also possessed several genes encoding proteins containing putative adhesion domains such as fibronectin-binding domains, MucBP domains, Cna protein B-type domain (Cna_B), von Willebrand factor type A domain (VWA), chitin-binding domain, and collagen binding domain. In particular, seven singleton genes encoding cell surface components were found (Table S10). One of these genes codes was for Cna protein B-type domain (CYU10_0CYU10_001640) that might improve cell attachment to collagen, a major component of extracellular matrix proteins in the intestine [55].

The improved growth of olive flounder fed with strain WFLU12 in our previous study [6] might be due to increased activities of enzymes such as *N*-acetyl-β-glucosaminidase and α-mannosidase in the gut. In this study, most *L. lactis* strains harbored genes encoding GH18 and GH38 (Table S3) responsible for yielding *N*-acetyl-β-glucosaminidase and α-mannosidase, respectively. In addition, two genes encoding thiolases found in weight gain-associated *Lactobacillus* spp. [56] were present in *L. lactis* genomes (one core gene, one dispensable gene) (Table S11). Thiolase plays key roles in various biosynthetic pathways such as fatty acid degradation through β-oxidation pathway and poly β-hydroxybutyric acid synthesis or steroid biogenesis [57]. This indicates that *L. lactis* could potentially participate in lipid digestion in the intestine and mobilize energy and carbon stored in fatty acids [56]. Indeed, our recent results showed that concentration of 3-hydroxybutyric acid in the gut was significantly higher in fish fed with probiotics than that in fish fed with the control diet (data not shown), indicating that these probiotic genes might be involved in metabolism improvement in the host gut.

Vitamin biosynthesis in *L. lactis* has been previously described [43,58,59]. In this study, we found that strain WFLU12 possessed a list of genes involved in metabolism of cofactors and vitamins (Table S11), including folate (vitamin B11), riboflavin (vitamin B2), and thiamine. These features of strain WFLU12 indicated that they might confer fish metabolic versatility in the conversion of material and energy in the form of carbohydrates, fats, and proteins in fish intestine. These data support the hypothesis that fish growth with probiotic additive might be associated with better dietary energy extraction and metabolism.

To compare potential probiotic properties of these 20 lactococcal strains used in this study, we generated a hierarchical clustering (Figure 6) of dispensable genes and assessed their potential involvement in probiotic effects. Strains originally isolated from a dairy source clearly showed different profiles in terms of bacteriocin, stress response, vitamin, and bacterial cell surface molecules, indicating the importance of industrial selection of probiotic candidates. In addition, strains CV56, SO, and IO-01 might be probiotic candidates as they are efficient producers of nisin. However, further work is needed to elucidate whether these identified genes are differentially expressed in the gut environment of the host. Based on genes observed in the genome of strain WFLU12 and our preliminary metabolite data detected in the gut of fish having dietary supplementation with strain WFLU12, we believe that *L. lactis* might be involved in increased metabolites such as nicotinamide and 3-hydroxybutyric acid (data not shown). Genetically, *L. lactis* only possesses genes involved in salvage NAD synthesis from nicotinamide (*deoD*), nicotinic acid (*deoD*, *pncB*), nicotinamide riboside (*nadD*), nicotinic acid riboside (*pncB*), and deamino-NAD+ (*nadD*, *nadE*) (Table S11). This indicates that *L. lactis* may depend on the availability of nicotinic acid from the environment to synthesize essential cofactor NAD. Thus, supplementation of this probiotic may limit nicotinic acid for pathogenic species, including Streptococci and Staphylococci that only have the salvage NAD pathway [60,61]. In addition, a short chain fatty acid (SCFA) is able to increase intracellular concentration of protons after entering and dissociating in more alkaline cytoplasm of bacterial cells. Therefore, growth of these cells might be inhibited since they have to spend energy to maintain intracellular pH at an optimal level [62,63]. Similar to SCFA, 3-hydroxybutyric acid (a storage compound of SCFA) is used as an alternative to antibiotics for the control of bacterial disease in aquaculture [63,64,65]. In our study, a significant increase in 3-hydroxybutyric acid in the gut of fish fed with dietary probiotic strain WFLU12 is believed to be related to the presence of two genes encoding thiolase (Table S11). Thus, this metabolite might contribute to the antimicrobial activity of this strain. It might be more efficient than using poly-hydroxybutyrate (PHB) particles in diets because the presence of much smaller sized PHB inside bacteria may improve their beneficial effects [63]. In general, strain WFLU12 possesses genes involved in producing compounds that may have growth-inhibitory effects on pathogenic bacteria. A further understanding of functions of these genes related to fish health is needed because some metabolism pathways are found to be limited or completely absent in fish due to the lack of host enzymes (for example, limited de novo synthesis of arginine in fish [66], lack of de novo synthesis of nucleotide in intestinal cells [67]). Thus, molecular mode of action of probiotics needs to be determined in more details.

## 3. Materials and Methods

### 3.1. Bacterial Genome Extraction and Sequencing

Genomic DNA was extracted from strain WFLU12 that was isolated from a wild olive flounder [6] using a QIAamp DNA Mini Kit (Qiagen, Hilden, Germany). The concentration of genomic DNA was measured by Qubit 2.0 Fluorometry (Thermo Fisher Scientific, Waltham, MA, USA). The purity of DNA samples (UV A260/A280) was assessed by NanoVue Plus™ spectrophotometry (GE Healthcare Life Sciences, Chicago, IL, USA). Sequencing the whole genome of the WFLU12 strain involved Nextera XT library prep, Illumina HiSeq 2500 paired-end sequencing with 500 MB data guaranteed, PacBio 10 kb genomic library preparation, and PacBio sequencing using 1 SMRT cell.

### 3.2. Genome Analysis, Assembly and Annotation

Using a combination of Illumina and PacBio reads, sequences were subjected to de novo Illumina/PacBio hybrid assembly and genome annotation. Scaffolds were subsequently marked using an in-house pipeline (BaseClear annotation pipeline) based on Prokka framework. Genome annotation was performed for the assembled contig or scaffold sequences using an annotation pipeline based on Prokka prokaryotic genome annotation system (http://vicbioinformatics.com/). The pipeline (based on Prokka version 1.6) included a number of features, including Prokaryote gene prediction by Prodigal v2, rRNA search using barrnap v0.2, tRNA prediction by Aragorn v1.2.36, pCDS physic-chemical properties using an in-house script, inferred proteins (EC number, CAZY number, and function annotation from UniProt BLAST best hit), signal peptide and cellular localization from signalp v4.0, conserved domains by hmmer-3, and Organism prediction using an in-house script.

This Whole Genome Shotgun project has been deposited at DDBJ/ENA/GenBank under the accession PKRZ00000000. The version described in this paper is version PKRZ01000000.

### 3.3. Genome Identification and Phylogenomic Analysis

To establish the phylogenomic identity of strain WFLU12, average nucleotide identity (ANI) was calculated with phylogenetic representatives of close hitherto-sequenced lactococcal genomes obtained from NCBI bacterial genome database (ftp://ftp.ncbi.nih.gov/genomes/) using JSpecies v1.2.1 [68] and visualized as heat map using Gene-e software (http://www.broadinstitute.org/cancer/software/GENE-E/).

To determine phylogenomic relationships of *L. lactis* subsp. *lactis* isolated from different sources, whole-genome phylogeny and hierarchical clustering were performed based on numerous orthologous genes and comparative gene content, respectively. A phylogenetic comparative approach based on thousands of orthologous genes in the core genome was conducted because it could give greater accuracy with a highly resolved phylogenetic tree and clarification of the phylogeny than analysis based on partial knowledge of DNA sequences [22]. It is known that hierarchical clustering analysis of relatively shared gene content between genomes can display important differences of biological evolution and metabolic reconstructions [21]. Using EDGAR v2.2 [22], both orthologous genes (1415 genes) and relatively shared gene content (2620 genes) between selected *L. lactis* strains were retrieved and a phylogenetic tree was generated. A hierarchical clustering tree was constructed using Genesis v1.8.1 [21].

### 3.4. Carbohydrate-Active Enzymes (Cazy) Profiles

To annotate carbohydrate-active enzymes, annotated protein dataset of each strain was retrieved from the NCBI bacterial genome database (ftp://ftp.ncbi.nih.gov/genomes/) and analyzed with dbCAN annotation server (http://csbl.bmb.uga.edu/dbCAN/annotate.php) [69]. These results were grouped into functional classes of glycoside hydrolases (GH), polysaccharide lyases (PL), carbohydrate esterases (CE), carbohydrate-binding modules (CBM), glycosyl transferases (GT), and auxiliary activities (AA). A heat map illustrating the distribution and abundance of CAZy numbers across tested strains was generated using software Gene-E.

### 3.5. Pan-Genome Analysis

Comparative pan-genome analysis of hitherto-sequenced *L. lactis* subsp. lactis strains including strain WFLU12 was performed with EDGAR v2.2 [22]. Accordingly, genomic subsets, including the number of core genome and singletons (strain-specific) in the gene pool, were extracted to understand the estimation of tracing horizontal gene-flux across strains and obtain insights into their evolution.

For functional analyses, genomic subsets within the pan-genome were subjected to the Clusters of Orthologous Group (COG) category-wise classification scheme by performing PSI-BLAST against the NCBI COG database (recently updated) in WebMGA analysis server using default parameters [70].

For analysis of the cell wall in relation to adhesive genes in the genome, automatic pilus cluster was searched using LOCP v.1.0.0 with default input parameters (*p*-value threshold of 1, p_adj-value threshold of 0.05) [71]. LOCP output results were then curated and the program was run on amino-acid CDSs data with default input parameters.

## 4. Conclusions

In this study, we found that *L. lactis* WFLU12 possessed functional genes involved in the stress response to the gastrointestinal tract environment, bacteriocin production, and dietary energy extraction and metabolism, supporting our previous study [6]. Comparative genomic analysis using hitherto sequenced genomes of *L. lactis* strains including strain WFLU12 in this study revealed niche-specific differences between strains in terms of genes and gene clusters for carbohydrate metabolism, envelope biogenesis, and bacterial defense mechanisms (lantibiotic transport, lysozyme biosynthesis). This host gut-derived strain WFLU12 harbored many genes supporting its probiotic action. Our results also highlight comparative genomics as a very useful and powerful tool to select probiotic candidates and predict their effects.

## Supplementary Materials

The following materials are available online (http://www.mdpi.com/1660-3397/16/5/140/s1): Figure S1: Functional annotation of ortholog groups in different parts of pan-genome of strain WFLU12. (A) Distribution of ortholog groups, functionally annotated or not annotated using search in COG database for different parts of the pan-genome. (B) Scaled heat map showing the distribution of functional classes for different parts of pan-genome. Figure S2: Non-uniform CAZy profiles analysis of *L. lactis* strains. Numbers of enzyme modules in each genome are shown. Overrepresented (red) and underrepresented modules (blue) are depicted as number of repertoires. Each strain is color-coded as indicated in Figure 2. Table S1: List of coding sequence (CDS) in L. lactis WFLU12 annotated using Prokka. Table S2: Complete genomes and genomic features of *Lactococcus* species used in genomic comparisons. Table S3: Distribution of CAZymes in 20 *L. lactis* complete genomes. Table S4: List of 129 singleton genes extracted from WFLU12 based on pan-genome analysis. Table S5: Growth characteristics of *L. lactis* strains WFLU12 on carbohydrates. Table S6: Antibiotic susceptibility of strain WFLU12. Table S7: Antibiotic resistance genes retrieved from L. lactis WFLU12 genome. Table S8: Bacteriocins encoding genes of 20 *L. lactis* extracted from pan-genome annotation. Table S9: Genes of WFLU12 coding for proteins involved in acid stress and bile salt resistance. Table S10: Genes of WFLU12 coding for bacterial cell surface molecules. Table S11: Genes of WFLU12 coding for proteins potentially involved in lipid and vitamin metabolism.
