# Supplementary material for: Genome-Wide Comparison Reveals a Probiotic Strain Lactococcus lactis WFLU12 Isolated from the Gastrointestinal Tract of Olive Flounder (Paralichthys olivaceus) Harboring Genes Supporting Probiotic Action
Source: Mar Drugs. 2018 Apr 24;16(5):140. doi: 10.3390/md16050140 (PMC5983272; doi:10.3390/md16050140)
Supplement: Supplementary file 1 [file marinedrugs-16-00140-s001.zip › marinedrugs-272713 Supplementary information/Supplementary Tables (180405_2).docx]

**Table S2.** Complete genomes and genomic features of *Lactococcus* species used in this study

| **Parameters** | **Genome size (bp)** | **G+C (%)** | **Number of genes** | **Protein** | **rRNA** | **tRNAs** | **No. of IS elements** | **Prophage ^§^** | **No. of plasmids** | **ANI**  **values ^#^** | **NCBI Accession no.** | **Ecological niche** | **Reference** |
| --- | --- | --- | --- | --- | --- | --- | --- | --- | --- | --- | --- | --- | --- |
| ***Lactococcus lactis* subsp. *lactis*** | | |  |  |  |  |  |  |  |  |  |  |  |
| WFLU12* | 2,511,408 | 35,07 | 2,534 | 2,480 | 19 | 67 | 7 | 1 / 4 / 3 | 0 | 100 | PKRZ00000000 | Fish | This study |
| IO-01 | 2,421,471 | 35.1 | 2,342 | 2,230 | 19 | 64 | 13 | 0 / 2 / 0 | 0 | 98.26 | AP012281 | Drain water | [1] |
| AI-06 | 2,398,091 | 35 | 2,320 | 2,178 | 19 | 62 | 5 | 1 / 0 / 1 | 0 | 97.98 | CP009472 | Açaí palm | [2] |
| KF147 | 2,598,144 | 34.86 | 2,595 | 2,445 | 19 | 69 | 21 | 1 / 1 / 2 | 1 | 98.15 | CP001834 | Mung bean | [3] |
| NCDO 2118 | 2,554,693 | 34.86 | 2,545 | 2,382 | 19 | 67 | 15 | 0 / 1 / 3 | 1 | 98.14 | CP009054 | Frozen peas | [4] |
| A12 | 2,603,898 | 35.49 | 2,865 | 2,696 | 19 | 65 | 31 | 1 / 1 /2 | 4 | 98.23 | LT599049.1 | Sourdough | [5] |
| UC08 | 2,459,220 | 35 | 2522 | 2310 | 19 | 65 | 7 | 0 / 2 / 0 | 3 | 98.28 | CP015903.1 | Meat | [5] |
| UC11 | 2,530,360 | 35 | 2505 | 2307 | 19 | 65 | 7 | 0 / 2 / 0 | 6 | 98.26 | CP015904.1 | Meat | [5] |
| SO | 2,488,699 | 35.2 | 2,482 | 2,311 | 19 | 64 | 34 | 2 / 1 /3 | 0 | 98.11 | CP010050.1 | Raw milk | [6] |
| UC06 | 2,680,420 | 35.26 | 2692 | 2478 | 19 | 71 | 25 | 2 / 1 /3 | 3 | 98.13 | CP015902.1 | Dairy isolate | [5] |
| KLDS 4.0325 | 2,589,250 | 35.39 | 2,648 | 2,448 | 19 | 64 | 30 | 2 / 3 / 5 | 3 | 98.16 | CP006766.1 | Koumiss | [7] |
| 184 | 2,368,670 | 35.16 | 2426 | 2345 | 15 | 51 | 40 | 2 / 2 / 4 | 3 | 98.14 | CP015895.1 | Dairy isolate | [5] |
| UC063 | 2,542,030 | 35.32 | 2623 | 2417 | 19 | 64 | 47 | 1 / 1 / 5 | 5 | 98.2 | CP015905.1 | Dairy isolate | [5] |
| UL8 | 2,535,460 | 35.29 | 2542 | 2346 | 18 | 62 | 40 | 1 / 1 / 7 | 3 | 98.22 | CP015908.1 | Dairy isolate | [5] |
| UC77 | 2,607,670 | 35.26 | 2735 | 2538 | 22 | 72 | 63 | 1 / 0 / 7 | 2 | 98.18 | CP015906.1 | Dairy isolate | [5] |
| 229 | 2,634,130 | 35.19 | 2736 | 2522 | 16 | 59 | 63 | 0 / 0 / 6 | 5 | 98.12 | CP015896.1 | Dairy isolate | [5] |
| Il1403 | 2,365,589 | 35.3 | 2,406 | 2,277 | 19 | 63 | 49 | 0 / 1 / 6 | 0 | 98.16 | AE005176 | Cheese | [8] |
| C10 | 2,337,760 | 35.3 | 2384 | 2298 | 15 | 50 | 35 | 1 / 0 / 7 | 1 | 98.21 | CP015898.1 | Dairy isolate | [5] |
| 275 | 2,753,890 | 35.49 | 2836 | 2613 | 19 | 63 | 29 | 4 / 1 / 2 | 4 | 98.17 | CP015897.1 | Dairy isolate | [5] |
| CV56 | 2,399,458 | 35.09 | 2,533 | 2,378 | 19 | 65 | 24 | 1 / 3 / 3 | 5 | 98.23 | CP002365 | Human | [9] |
| ***Lactococcus* *lactis* subsp. *cremoris*** | | |  |  |  |  |  |  |  |  |  |  |  |
| KW2 | 2,427,048 | 35,70 | 2,353 | 2,268 | 19 | 61 |  |  | 0 | 88.01 | CP004884 | Corn | [10] |
| MG1363 | 2,529,478 | 35,70 | 2,597 | 2,434 | 19 | 64 |  |  | 0 | 88.59 | AM406671 | Dairy starter | [11] |
| NZ9000 | 2,530,294 | 35,70 | 2,594 | 2,510 | 19 | 64 |  |  | 0 | 88.62 | CP002094 | Dairy starter | [11] |
| ***Lactococcus piscium*** | |  |  |  |  |  |  |  |  |  |  |  |  |
| MKFS47 | 2,503,066 | 38.59 | 2489 | 2356 | 13 | 55 |  |  | 2 | 82.96 |  | Filet strips | [12] |
| ***Lactococcus garviae*** | |  |  |  |  |  |  |  |  |  |  |  |  |
| ATCC_49156 | 1,950,135 | 38,80 | 2,024 | 1,947 | 16 | 62 |  |  | 0 | 83.26 | AP009332 | Yellowtail | [13] |
| Lg2 | 1,963,964 | 38,80 | 2,045 | 1,968 | 16 | 62 |  |  | 0 | 83.6 | AP009333 | Yellowtail | [13] |
| *Lactococcus garvieae* are fish pathogens used as a closely related out group in the analyses.  §, prophage types include questionable prophage/incomplete prophage/ intact prophage (<http://phast.wishartlab.com>)  #, ANI analysis values of strain WFLU12 against and reference complete genomes. | | | | | | | | | | | | |  |

**Table S3.** Distribution of CAZymes in 20 *L. lactis* genomes

| **CAZy classes/ families** | **1** | **2** | **3** | **4** | **5** | **6** | **7** | **8** | **9** | **10** | **11** | **12** | **13** | **14** | **15** | **16** | **17** | **18** | **19** | **20** |
| --- | --- | --- | --- | --- | --- | --- | --- | --- | --- | --- | --- | --- | --- | --- | --- | --- | --- | --- | --- | --- |
| AA10 | 1 | 1 | 1 | 1 | 1 | 1 | 1 | 1 | 1 | 1 | 1 | 1 | 1 | 1 | 1 | 1 | 1 | 1 | 1 | 1 |
| AA3 | 1 | 1 | 1 | 1 | 1 | 1 | 1 | 1 | 1 | 0 | 1 | 1 | 1 | 1 | 1 | 1 | 1 | 0 | 1 | 1 |
| AA6 | 1 | 0 | 0 | 0 | 0 | 0 | 0 | 0 | 0 | 0 | 0 | 0 | 0 | 0 | 0 | 0 | 0 | 0 | 0 | 0 |
| CBM11 | 2 | 2 | 2 | 2 | 2 | 1 | 2 | 2 | 2 | 2 | 2 | 2 | 2 | 2 | 2 | 2 | 2 | 2 | 2 | 2 |
| CBM22 | 1 | 0 | 0 | 0 | 0 | 0 | 0 | 0 | 0 | 0 | 0 | 0 | 0 | 0 | 0 | 0 | 0 | 0 | 0 | 0 |
| CBM26 | 0 | 0 | 0 | 0 | 0 | 0 | 0 | 0 | 0 | 0 | 0 | 1 | 1 | 0 | 0 | 1 | 0 | 0 | 0 | 1 |
| CBM30 | 0 | 0 | 0 | 1 | 1 | 1 | 1 | 0 | 0 | 0 | 0 | 0 | 0 | 0 | 0 | 0 | 0 | 0 | 0 | 0 |
| CBM32 | 2 | 1 | 1 | 1 | 1 | 1 | 1 | 0 | 1 | 1 | 1 | 1 | 1 | 1 | 1 | 1 | 1 | 1 | 0 | 1 |
| CBM34 | 2 | 2 | 2 | 2 | 2 | 2 | 2 | 2 | 2 | 2 | 2 | 2 | 2 | 2 | 2 | 2 | 2 | 2 | 2 | 2 |
| CBM37 | 0 | 1 | 0 | 1 | 0 | 0 | 0 | 0 | 0 | 0 | 0 | 0 | 0 | 0 | 0 | 0 | 0 | 0 | 0 | 0 |
| CBM38 | 0 | 0 | 0 | 0 | 0 | 0 | 0 | 0 | 0 | 0 | 0 | 0 | 0 | 1 | 1 | 0 | 0 | 0 | 0 | 0 |
| CBM39 | 0 | 0 | 0 | 0 | 0 | 0 | 0 | 1 | 0 | 0 | 0 | 0 | 0 | 0 | 0 | 0 | 0 | 0 | 0 | 0 |
| CBM44 | 0 | 0 | 0 | 0 | 0 | 0 | 0 | 0 | 1 | 1 | 0 | 1 | 1 | 1 | 1 | 1 | 1 | 1 | 0 | 1 |
| CBM48 | 1 | 1 | 1 | 1 | 1 | 1 | 1 | 1 | 0 | 1 | 1 | 1 | 1 | 1 | 1 | 1 | 1 | 1 | 1 | 1 |
| CBM5 | 1 | 1 | 1 | 1 | 1 | 1 | 1 | 0 | 1 | 1 | 1 | 1 | 3 | 1 | 1 | 1 | 1 | 3 | 1 | 1 |
| CBM50 | 10 | 9 | 10 | 8 | 7 | 7 | 11 | 8 | 8 | 9 | 7 | 11 | 10 | 12 | 10 | 8 | 12 | 9 | 8 | 8 |
| CBM56 | 0 | 0 | 0 | 0 | 0 | 0 | 1 | 0 | 0 | 0 | 1 | 0 | 0 | 0 | 0 | 0 | 0 | 0 | 0 | 0 |
| CBM58 | 0 | 0 | 0 | 0 | 1 | 1 | 0 | 0 | 0 | 0 | 2 | 0 | 0 | 0 | 0 | 0 | 0 | 0 | 0 | 0 |
| CBM60 | 1 | 1 | 1 | 1 | 1 | 1 | 1 | 1 | 1 | 1 | 1 | 1 | 1 | 1 | 1 | 1 | 1 | 0 | 1 | 1 |
| CBM66 | 1 | 0 | 0 | 0 | 0 | 0 | 0 | 0 | 0 | 0 | 0 | 0 | 0 | 0 | 0 | 0 | 0 | 0 | 0 | 0 |
| CE1 | 4 | 4 | 4 | 4 | 3 | 3 | 3 | 3 | 3 | 3 | 3 | 3 | 3 | 3 | 3 | 4 | 3 | 3 | 3 | 3 |
| CE10 | 3 | 2 | 3 | 3 | 3 | 3 | 2 | 3 | 3 | 3 | 3 | 3 | 2 | 3 | 3 | 2 | 3 | 3 | 2 | 3 |
| CE12 | 1 | 1 | 1 | 1 | 1 | 1 | 1 | 1 | 1 | 1 | 1 | 2 | 1 | 1 | 1 | 0 | 1 | 1 | 1 | 1 |
| CE3 | 2 | 3 | 3 | 3 | 2 | 2 | 2 | 2 | 2 | 1 | 2 | 2 | 2 | 2 | 2 | 2 | 2 | 2 | 2 | 2 |
| CE4 | 4 | 3 | 3 | 4 | 4 | 5 | 4 | 4 | 3 | 2 | 4 | 3 | 3 | 3 | 4 | 4 | 3 | 3 | 3 | 3 |
| CE6 | 2 | 2 | 2 | 2 | 2 | 2 | 2 | 0 | 0 | 0 | 1 | 0 | 0 | 0 | 0 | 2 | 0 | 0 | 0 | 0 |
| CE7 | 1 | 1 | 1 | 1 | 1 | 1 | 0 | 1 | 0 | 0 | 1 | 0 | 0 | 0 | 0 | 0 | 0 | 0 | 1 | 0 |
| CE9 | 1 | 1 | 1 | 1 | 1 | 1 | 1 | 1 | 1 | 1 | 1 | 1 | 1 | 1 | 1 | 1 | 1 | 1 | 1 | 0 |
| GH1 | 7 | 9 | 9 | 9 | 8 | 8 | 6 | 7 | 6 | 7 | 9 | 7 | 7 | 6 | 6 | 4 | 6 | 7 | 8 | 6 |
| GH105 | 1 | 0 | 0 | 0 | 0 | 0 | 0 | 0 | 0 | 0 | 0 | 0 | 0 | 0 | 0 | 0 | 0 | 0 | 0 | 0 |
| GH109 | 4 | 5 | 5 | 5 | 5 | 5 | 4 | 4 | 4 | 4 | 4 | 4 | 4 | 4 | 4 | 5 | 4 | 4 | 4 | 4 |
| GH113 | 1 | 1 | 1 | 1 | 1 | 1 | 0 | 1 | 1 | 1 | 1 | 0 | 0 | 0 | 0 | 0 | 0 | 1 | 1 | 0 |
| GH115 | 0 | 1 | 1 | 0 | 0 | 0 | 0 | 1 | 0 | 1 | 0 | 0 | 0 | 0 | 0 | 0 | 0 | 0 | 0 | 0 |
| GH125 | 1 | 1 | 1 | 1 | 1 | 1 | 1 | 1 | 1 | 0 | 1 | 1 | 1 | 1 | 1 | 1 | 1 | 1 | 1 | 1 |
| GH13 | 8 | 9 | 8 | 8 | 8 | 8 | 8 | 8 | 7 | 8 | 8 | 9 | 7 | 8 | 8 | 8 | 8 | 8 | 10 | 8 |
| GH18 | 1 | 1 | 1 | 1 | 1 | 1 | 1 | 0 | 1 | 1 | 1 | 1 | 2 | 1 | 1 | 1 | 1 | 2 | 1 | 1 |
| GH2 | 0 | 1 | 1 | 1 | 1 | 1 | 0 | 1 | 1 | 1 | 1 | 1 | 1 | 1 | 1 | 1 | 1 | 1 | 1 | 1 |
| GH20 | 1 | 1 | 1 | 1 | 1 | 1 | 1 | 1 | 1 | 1 | 1 | 1 | 1 | 1 | 1 | 1 | 1 | 1 | 1 | 1 |
| GH23 | 1 | 1 | 2 | 1 | 1 | 1 | 1 | 1 | 2 | 3 | 1 | 2 | 1 | 3 | 3 | 2 | 1 | 2 | 1 | 1 |
| GH24 | 1 | 0 | 0 | 0 | 0 | 0 | 0 | 0 | 0 | 0 | 0 | 0 | 0 | 0 | 0 | 0 | 0 | 0 | 0 | 0 |
| GH25 | 4 | 5 | 5 | 2 | 3 | 3 | 4 | 2 | 1 | 2 | 2 | 3 | 3 | 4 | 3 | 2 | 4 | 1 | 2 | 2 |
| GH28 | 1 | 0 | 0 | 0 | 0 | 0 | 0 | 0 | 0 | 0 | 0 | 0 | 0 | 0 | 0 | 0 | 0 | 0 | 0 | 0 |
| GH3 | 1 | 1 | 1 | 1 | 1 | 1 | 1 | 1 | 1 | 1 | 1 | 1 | 1 | 1 | 1 | 1 | 1 | 1 | 1 | 1 |
| GH31 | 1 | 0 | 0 | 0 | 0 | 0 | 0 | 0 | 0 | 0 | 0 | 1 | 1 | 1 | 1 | 1 | 1 | 0 | 1 | 0 |
| GH32 | 1 | 1 | 1 | 1 | 1 | 1 | 1 | 1 | 0 | 0 | 0 | 0 | 0 | 0 | 0 | 0 | 0 | 0 | 0 | 1 |
| GH35 | 1 | 0 | 0 | 0 | 0 | 0 | 0 | 0 | 0 | 0 | 0 | 0 | 0 | 0 | 0 | 0 | 0 | 0 | 0 | 0 |
| GH36 | 0 | 1 | 0 | 0 | 0 | 0 | 0 | 0 | 0 | 0 | 0 | 0 | 0 | 0 | 0 | 0 | 0 | 0 | 2 | 0 |
| GH38 | 1 | 2 | 2 | 1 | 1 | 1 | 1 | 1 | 1 | 1 | 1 | 1 | 1 | 1 | 1 | 1 | 1 | 1 | 1 | 1 |
| GH4 | 0 | 0 | 0 | 0 | 0 | 0 | 0 | 0 | 0 | 0 | 1 | 0 | 0 | 0 | 0 | 0 | 0 | 0 | 0 | 0 |
| GH43 | 1 | 3 | 3 | 3 | 1 | 1 | 1 | 1 | 1 | 1 | 1 | 1 | 1 | 1 | 1 | 1 | 1 | 1 | 1 | 1 |
| GH65 | 3 | 2 | 2 | 2 | 4 | 2 | 2 | 2 | 2 | 2 | 2 | 2 | 2 | 2 | 2 | 2 | 2 | 2 | 3 | 2 |
| GH67 | 1 | 1 | 1 | 1 | 1 | 1 | 1 | 1 | 0 | 0 | 1 | 0 | 0 | 0 | 0 | 1 | 0 | 0 | 0 | 0 |
| GH73 | 4 | 4 | 4 | 4 | 5 | 5 | 4 | 4 | 4 | 4 | 4 | 5 | 4 | 4 | 4 | 4 | 4 | 4 | 3 | 6 |
| GH77 | 1 | 1 | 1 | 1 | 1 | 1 | 1 | 1 | 1 | 1 | 1 | 1 | 1 | 1 | 1 | 1 | 1 | 1 | 1 | 1 |
| GH78 | 0 | 0 | 0 | 0 | 1 | 1 | 0 | 0 | 0 | 0 | 0 | 0 | 0 | 0 | 0 | 0 | 0 | 0 | 0 | 0 |
| GH8 | 2 | 2 | 2 | 1 | 1 | 1 | 2 | 1 | 0 | 0 | 1 | 0 | 0 | 0 | 0 | 1 | 0 | 0 | 0 | 0 |
| GH85 | 1 | 1 | 1 | 1 | 1 | 1 | 1 | 0 | 1 | 1 | 1 | 1 | 1 | 1 | 1 | 1 | 1 | 1 | 0 | 1 |
| GH88 | 1 | 0 | 0 | 0 | 0 | 0 | 0 | 0 | 0 | 0 | 0 | 0 | 0 | 0 | 0 | 0 | 0 | 0 | 0 | 0 |
| GH9 | 0 | 0 | 0 | 0 | 0 | 0 | 1 | 0 | 0 | 0 | 0 | 0 | 0 | 0 | 0 | 0 | 0 | 0 | 0 | 0 |
| GH92 | 1 | 1 | 1 | 1 | 1 | 1 | 1 | 1 | 1 | 1 | 1 | 1 | 1 | 1 | 1 | 1 | 1 | 1 | 0 | 1 |
| GT11 | 0 | 1 | 1 | 0 | 0 | 0 | 0 | 0 | 0 | 0 | 0 | 0 | 0 | 0 | 0 | 0 | 0 | 0 | 1 | 0 |
| GT19 | 1 | 1 | 1 | 1 | 1 | 1 | 1 | 1 | 1 | 1 | 1 | 1 | 1 | 1 | 1 | 1 | 1 | 1 | 1 | 1 |
| GT2 | 13 | 17 | 17 | 20 | 14 | 15 | 13 | 15 | 12 | 12 | 13 | 16 | 16 | 15 | 15 | 13 | 13 | 13 | 15 | 14 |
| GT28 | 1 | 1 | 1 | 2 | 1 | 1 | 1 | 1 | 1 | 1 | 1 | 2 | 1 | 1 | 1 | 1 | 1 | 1 | 1 | 1 |
| GT32 | 0 | 0 | 0 | 1 | 1 | 1 | 0 | 0 | 0 | 0 | 0 | 1 | 0 | 0 | 0 | 1 | 1 | 1 | 0 | 0 |
| GT35 | 1 | 1 | 1 | 1 | 1 | 1 | 1 | 1 | 1 | 1 | 1 | 1 | 1 | 1 | 1 | 1 | 1 | 1 | 1 | 1 |
| GT4 | 3 | 8 | 8 | 8 | 8 | 7 | 5 | 6 | 7 | 7 | 7 | 7 | 8 | 8 | 8 | 10 | 11 | 9 | 8 | 6 |
| GT5 | 1 | 1 | 1 | 1 | 1 | 1 | 1 | 1 | 1 | 1 | 1 | 1 | 1 | 1 | 1 | 1 | 1 | 1 | 1 | 1 |
| GT51 | 3 | 3 | 3 | 3 | 3 | 3 | 3 | 3 | 3 | 3 | 3 | 3 | 3 | 3 | 3 | 3 | 3 | 3 | 3 | 3 |
| GT66 | 0 | 0 | 0 | 0 | 0 | 0 | 1 | 0 | 0 | 0 | 0 | 0 | 0 | 0 | 0 | 0 | 0 | 0 | 0 | 0 |
| GT8 | 0 | 0 | 0 | 0 | 0 | 0 | 1 | 1 | 0 | 0 | 0 | 0 | 0 | 0 | 0 | 0 | 0 | 0 | 0 | 0 |
| GT92 | 0 | 1 | 1 | 0 | 0 | 0 | 0 | 0 | 0 | 0 | 0 | 0 | 0 | 0 | 0 | 0 | 0 | 1 | 0 | 0 |
| PL8 | 1 | 0 | 0 | 0 | 0 | 0 | 0 | 0 | 0 | 0 | 0 | 0 | 0 | 0 | 0 | 0 | 0 | 0 | 0 | 0 |
| CAZy families and copy numbers were computed by the dbCAN (http://csbl.bmb.uga.edu/dbCAN/annotate.php) annotation server.  1, WFLU12; 2, KF147; 3, NCDO_2118; 4, AI_06; 5, UC08; 6, UC11; 7, IO–01; 8, SO; 9, IL1403; 10, UC77; 11, UC06; 12, 275; 13, UC63; 14, C10; 15, UL8; 16, KLDS40325; 17, 184; 18, 229; 19, A12; 20, CV56 | | | | | | | | | | | | | | | | | | | | |

**Table S4.** List of 129 singleton genes extracted from *L. lactis* WFLU12 based on pan-genome analysis

| **Locus_tag** | **Start** | **End** | **Gene** | **Cellular_localization** | **EC_number** | **CaZy** | **UniProtKB_product_1** |
| --- | --- | --- | --- | --- | --- | --- | --- |
| CYU10_000003 | 1758 | 3254 | SP_1800 | Unknown | - | - | Putative trans-acting regulator SP_1800 |
| CYU10_000004 | 3730 | 3266 | - | Unknown | - | - | - |
| CYU10_000005 | 4657 | 4052 | - | Cytoplasmic | - | - | - |
| CYU10_000006 | 5879 | 5034 | - | Cytoplasmic | - | - | - |
| CYU10_000007 | 6694 | 6071 | yoqW | Unknown | 3.4.-.- | - | Putative SOS response-associated peptidase yoqW |
| CYU10_000008 | 7485 | 7216 | - | Cytoplasmic | - | - | - |
| CYU10_000037 | 38717 | 37881 | - | Cell wall | - | - | - |
| CYU10_000081 | 84422 | 84646 | - | Unknown | - | - | - |
| CYU10_000141 | 145350 | 144922 | - | Cytoplasmic | 2.3.1.82 | - | Aminoglycoside N(6')-acetyltransferase type 1 |
| CYU10_000143 | 147613 | 147047 | - | Unknown | - | - | - |
| CYU10_000144 | 148128 | 147604 | - | Cytoplasmic | - | - | - |
| CYU10_000154 | 152799 | 152560 | - | Cytoplasmic | - | - | - |
| CYU10_000157 | 156003 | 155557 | 1 | Cytoplasmic | - | - | Terminase small subunit |
| CYU10_000158 | 157419 | 156274 | L3_1 | Unknown | 3.2.1.17 | GH24 | Lysozyme |
| CYU10_000164 | 159722 | 160189 | - | Cytoplasmic Membrane | - | - | - |
| CYU10_000175 | 165326 | 165511 | - | Unknown | - | - | - |
| CYU10_000176 | 165717 | 165508 | - | Unknown | - | - | - |
| CYU10_000177 | 165872 | 166477 | - | Cytoplasmic Membrane | - | - | - |
| CYU10_000292 | 291005 | 290748 | - | Cytoplasmic | - | - | - |
| CYU10_000317 | 310240 | 309977 | - | Cytoplasmic | - | - | - |
| CYU10_000319 | 310780 | 310604 | - | Unknown | - | - | - |
| CYU10_000322 | 311592 | 311440 | - | Unknown | - | - | - |
| CYU10_000330 | 316885 | 316433 | - | Cytoplasmic | - | - | - |
| CYU10_000333 | 319470 | 318961 | - | Cytoplasmic | - | - | - |
| CYU10_000342 | 323609 | 323505 | - | Unknown | - | - | - |
| CYU10_000535 | 525805 | 525194 | - | Unknown | - | - | - |
| CYU10_000536 | 525969 | 525802 | - | Unknown | - | - | - |
| CYU10_000538 | 527536 | 526649 | L3_2 | Unknown | 3.2.1.17 | GH25 | Lysozyme |
| CYU10_000541 | 528374 | 528117 | - | Cytoplasmic | - | - | - |
| CYU10_000559 | 549172 | 548624 | - | Cytoplasmic | - | - | - |
| CYU10_000561 | 550609 | 550358 | - | Cytoplasmic Membrane | - | - | - |
| CYU10_000569 | 554990 | 554565 | - | Cytoplasmic | - | - | - |
| CYU10_000570 | 555307 | 554981 | - | Cytoplasmic | - | - | - |
| CYU10_000571 | 555809 | 555318 | - | Cytoplasmic | - | - | - |
| CYU10_000575 | 557077 | 557454 | HI_1406 | Cytoplasmic | - | - | Uncharacterized protein HI_1406 |
| CYU10_000584 | 560502 | 560801 | - | Cytoplasmic | - | - | - |
| CYU10_000646 | 617444 | 615708 | - | Cytoplasmic | - | - | - |
| CYU10_000647 | 617676 | 617431 | - | Cytoplasmic | - | - | - |
| CYU10_000648 | 618622 | 617642 | - | Cytoplasmic | - | - | - |
| CYU10_000651 | 619898 | 619470 | - | Cytoplasmic | 2.3.1.82 | - | Aminoglycoside N(6')-acetyltransferase type 1 |
| CYU10_000652 | 621142 | 620603 | - | Cytoplasmic | - | - | - |
| CYU10_000664 | 629338 | 629186 | - | Cytoplasmic | - | - | - |
| CYU10_000719 | 678821 | 679672 | MJ0912 | Cytoplasmic | - | - | Uncharacterized protein MJ0912 |
| CYU10_000729 | 685073 | 684849 | - | Cytoplasmic | - | - | - |
| CYU10_000735 | 687047 | 687595 | - | Cytoplasmic | - | - | - |
| CYU10_000736 | 687798 | 688661 | - | Unknown | - | - | - |
| CYU10_000737 | 688842 | 689048 | - | Cytoplasmic Membrane | - | - | - |
| CYU10_000738 | 689887 | 690318 | - | Cytoplasmic Membrane | - | - | - |
| CYU10_000742 | 692680 | 693039 | - | Cytoplasmic Membrane | - | - | - |
| CYU10_000856 | 796060 | 795467 | yxbB | Cytoplasmic | - | - | Uncharacterized protein YxbB |
| CYU10_000921 | 865943 | 864255 | YPL245W | Unknown | - | - | Uncharacterized protein YPL245W |
| CYU10_000992 | 936057 | 935389 | srtF_1 | Cytoplasmic Membrane | - | - | Lantibiotic transport ATP-binding protein SrtF |
| CYU10_000993 | 937063 | 936047 | - | Cytoplasmic Membrane | - | - | - |
| CYU10_001153 | 1088452 | 1089090 | - | Unknown | - | - | - |
| CYU10_001154 | 1089174 | 1089713 | - | Cytoplasmic | - | - | - |
| CYU10_001161 | 1103476 | 1102781 | - | Unknown | - | - | - |
| CYU10_001215 | 1153320 | 1155098 | - | Cytoplasmic Membrane | - | - | - |
| CYU10_001221 | 1161446 | 1161339 | - | Unknown | - | - | - |
| CYU10_001246 | 1183289 | 1183930 | - | Cytoplasmic Membrane | - | - | - |
| CYU10_001268 | 1203373 | 1203669 | - | Cytoplasmic Membrane | - | - | - |
| CYU10_001477 | 1417930 | 1418739 | nhoA | Cytoplasmic | 2.3.1.118 | - | N-hydroxyarylamine O-acetyltransferase |
| CYU10_001633 | 1564013 | 1564201 | - | Cytoplasmic Membrane | - | - | - |
| CYU10_001634 | 1564729 | 1564517 | - | Unknown | - | - | - |
| CYU10_001635 | 1564918 | 1564778 | - | Extracellular | - | - | zinc-responsive transcriptional regulator |
| CYU10_001636 | 1565363 | 1565608 | - | Unknown | - | - | - |
| CYU10_001640 | 1571157 | 1572650 | - | Cell wall | - | - | Fimbrial subunit type 2 |
| CYU10_001641 | 1572756 | 1573979 | - | Unknown | - | - | - |
| CYU10_001706 | 1630067 | 1630303 | - | Cytoplasmic Membrane | - | - | - |
| CYU10_001707 | 1630853 | 1630296 | - | Unknown | - | - | - |
| CYU10_001797 | 1729385 | 1730164 | - | Unknown | - | - | - |
| CYU10_001923 | 1866335 | 1866670 | - | Unknown | - | - | - |
| CYU10_001924 | 1866676 | 1867170 | - | Unknown | - | - | - |
| CYU10_001926 | 1868738 | 1870501 | - | Cytoplasmic Membrane | - | - | - |
| CYU10_001928 | 1872400 | 1872684 | - | Unknown | - | - | - |
| CYU10_001984 | 1924971 | 1927763 | ggaB_1 | Cytoplasmic Membrane | - | GT2 | Minor teichoic acid biosynthesis protein GgaB |
| CYU10_001985 | 1927738 | 1928754 | - | Cytoplasmic | - | - | - |
| CYU10_001986 | 1928747 | 1930336 | ggaB_2 | Unknown | - | GT2 | Minor teichoic acid biosynthesis protein GgaB |
| CYU10_001987 | 1930538 | 1931632 | tarL | Cytoplasmic Membrane | 2.7.8.14 | - | Putative polyribitolphosphotransferase |
| CYU10_001990 | 1933450 | 1934823 | - | Unknown | - | - | - |
| CYU10_002145 | 2089859 | 2090140 | - | Cytoplasmic Membrane | - | - | - |
| CYU10_002146 | 2090198 | 2091355 | - | Unknown | - | - | - |
| CYU10_002199 | 2148564 | 2148271 | - | Unknown | - | - | - |
| CYU10_002200 | 2150282 | 2148759 | SP_0314 | Extracellular | 4.2.2.1 | PL8 | Hyaluronate lyase |
| CYU10_002201 | 2151248 | 2150424 | - | Unknown | - | - | - |
| CYU10_002202 | 2151609 | 2152763 | ugl | Cytoplasmic | 3.2.1.180 | GH88 | Unsaturated chondroitin disaccharide hydrolase |
| CYU10_002203 | 2152773 | 2154566 | Glb1l2 | Cytoplasmic | 3.2.1.- | GH35 | Beta-galactosidase-1-like protein 2 |
| CYU10_002204 | 2155283 | 2154576 | dasR | Unknown | - | - | HTH-type transcriptional repressor DasR |
| CYU10_002205 | 2155512 | 2156441 | manX_2 | Cytoplasmic | 2.7.1.69 | - | Mannose-specific phosphotransferase enzyme IIB component |
| CYU10_002206 | 2156444 | 2157241 | sorA_2 | Cytoplasmic Membrane | - | - | Sorbose permease IIC component |
| CYU10_002207 | 2157234 | 2158058 | agaD | Cytoplasmic Membrane | - | - | N-acetylgalactosamine permease IID component |
| CYU10_002208 | 2158358 | 2159194 | - | Unknown | - | - | - |
| CYU10_002209 | 2159251 | 2161029 | - | Cytoplasmic Membrane | - | - | - |
| CYU10_002211 | 2162550 | 2163395 | kduI2 | Cytoplasmic | 5.3.1.17 | - | 4-deoxy-L-threo-5-hexosulose-uronate ketol-isomerase 2 |
| CYU10_002212 | 2163432 | 2164238 | idnO | Cytoplasmic | 1.1.1.69 | - | Gluconate 5-dehydrogenase |
| CYU10_002215 | 2166923 | 2165946 | rhaR | Cytoplasmic | - | - | HTH-type transcriptional activator RhaR |
| CYU10_002216 | 2167213 | 2168649 | yjmB_1 | Cytoplasmic Membrane | - | - | Uncharacterized symporter YjmB |
| CYU10_002217 | 2168677 | 2168913 | - | Unknown | - | - | - |
| CYU10_002218 | 2168936 | 2170054 | yesR | Cytoplasmic | 3.2.1.172 | - | Unsaturated rhamnogalacturonyl hydrolase YesR |
| CYU10_002219 | 2170070 | 2170900 | - | Cytoplasmic | - | - | - |
| CYU10_002220 | 2171113 | 2172417 | GSVIVT  00026920001 | Cytoplasmic | 3.2.1.15 | - | Probable polygalacturonase |
| CYU10_002221 | 2172514 | 2173965 | rhaB | Unknown | 2.7.1.5 | - | Rhamnulokinase |
| CYU10_002222 | 2173992 | 2175254 | rhaA | Cytoplasmic | 5.3.1.14 | - | L-rhamnose isomerase |
| CYU10_002223 | 2175349 | 2176218 | rhaD | Cytoplasmic | 4.1.2.19 | - | Rhamnulose-1-phosphate aldolase |
| CYU10_002224 | 2176229 | 2176543 | rhaM | Cytoplasmic | 5.1.3.32 | - | L-rhamnose mutarotase |
| CYU10_002225 | 2176573 | 2177712 | fucO | Cytoplasmic | 1.1.1.77 | - | Lactaldehyde reductase |
| CYU10_002236 | 2190043 | 2190438 | - | Unknown | - | - | - |
| CYU10_002237 | 2190655 | 2191992 | - | Extracellular | - | - | bacterial surface protein 26-residue repeat |
| CYU10_002251 | 2203333 | 2204073 | mutS_2 | Unknown | - | - | DNA mismatch repair protein MutS |
| CYU10_002269 | 2226388 | 2225693 | - | Unknown | - | - | - |
| CYU10_002299 | 2263135 | 2262986 | - | Unknown | - | - | - |
| CYU10_002311 | 3053 | 3262 | - | Cytoplasmic | - | - | - |
| CYU10_002312 | 3563 | 3192 | - | Unknown | - | - | - |
| CYU10_002316 | 4461 | 4883 | - | Cytoplasmic | - | - | - |
| CYU10_002318 | 5192 | 5374 | - | Cytoplasmic | - | - | - |
| CYU10_002319 | 5367 | 5774 | - | Cytoplasmic | - | - | - |
| CYU10_002320 | 5786 | 6031 | - | Unknown | - | - | - |
| CYU10_002323 | 7086 | 7928 | - | Unknown | - | - | - |
| CYU10_002324 | 7932 | 8111 | - | Cytoplasmic Membrane | - | - | - |
| CYU10_002328 | 10891 | 12525 | - | Unknown | - | - | - |
| CYU10_002332 | 14891 | 15247 | - | Unknown | - | - | - |
| CYU10_002333 | 15270 | 16277 | - | Unknown | - | - | - |
| CYU10_002335 | 16531 | 16872 | - | Cytoplasmic | - | - | - |
| CYU10_002344 | 25623 | 27338 | - | Cytoplasmic | - | - | - |
| CYU10_002345 | 27347 | 28102 | - | Unknown | - | - | - |
| CYU10_002349 | 30255 | 30701 | - | Cytoplasmic | - | - | - |
| CYU10_002350 | 30713 | 31177 | - | Unknown | - | - | - |
| CYU10_002351 | 31250 | 31122 | - | Unknown | - | - | - |
| CYU10_002385 | 65277 | 65498 | - | Extracellular | - | - | - |
| CYU10_002555 | 233935 | 233243 | - | Unknown | - | - | - |

**Table S5.** Growth characteristics of *L. lactis* WFLU12 on carbohydrates

| Carbohydrate | *L. lactis* WFLU12 | Carbohydrate type |
| --- | --- | --- |
| Glycerol | – |  |
| L–ARAbinose | – | Pentose |
| D–Ribose | + | Pentose |
| D–Xylose | + | Pentose |
| D–Galactose | + | Hexose |
| D–Glucose | + | Hexose |
| D–Fructose | + | Hexose |
| D–Mannose | + | Hexose |
| D–Mannitol | + | Hexose |
| L-Rhamnose | + | Hexose |
| N–AcetylGlucosamine | + | Hexose |
| Amygdalin | + | Aryl-disaccharide |
| Arbutin | + | Aryl-monosaccharide |
| Escutin ferric citrate | + | Aryl-monosaccharide |
| Salicin | + | Aryl-monosaccharide |
| D–Cellobiose | + | Disaccharide |
| D–Maltose | + | Disaccharide |
| D–Lactose | + | Disaccharide |
| D–Melibiose | + | Disaccharide |
| D–Saccharose | + | Disaccharide |
| D–Trehalose | + | Disaccharide |
| Gentiobiose | + | Disaccharide |
| Amidon (starch) | + | Polysaccharide |
| D–Sorbitol | + |  |
| D–Turanose | – | Disaccharide |
| Inulin | – |  |
| D–Melezitose | – | Trisaccharide |
| D–Raffinose | – | Trisaccharide |

**Table S6.** Antibiotic susceptibility of *L. lactis* WFLU12

| **Antibiotics** | **Conc.** | **Inhibition zone (mm)** | **Susceptibility*** |
| --- | --- | --- | --- |
| Ampicillin | AMP10 | 34 | S |
| Amoxicillin | AML25 | 38 | S |
| Doxycycline | D30 | 31 | S |
| Oxytetracycline | T30 | 30 | S |
| Erythromycin | E15 | 30 | S |
| Ciprofloxacin | CIP5 | 19 | S |
| Enrofloxacin | ENR5 | 28 | S |
| Florfenicol | FFC30 | 30 | S |
| Chloramphenicol | C30 | 29 | S |
| Cephalothin | KF30 | 28 | S |
| Cefuroxime | CXM30 | 38 | S |
| Cefoperazone | CFP75 | 31 | S |
| Cephalexin | CL30 | 15 | R |
| gentamicin | CN10 | 20 | S |
| Sulfamethoxazole /trimethoprim | SXT | 13 | R |
| Sulfadiazine | SD25 | 24 | S |
| Sulfamethoxazole | RL25 | 10 | R |
| *R=resistant, S=susceptible. | | | |

**Table S8** Bacteriocin encoding genes of 20 *L. lactis* extracted from pan genome annotation.

| Bacteriocin encoding gene | Strains | | | | | | | | | | | | | | | | | | | |
| --- | --- | --- | --- | --- | --- | --- | --- | --- | --- | --- | --- | --- | --- | --- | --- | --- | --- | --- | --- | --- |
|  | 1 | 2 | 3 | 4 | 5 | 6 | 7 | 8 | 9 | 10 | 11 | 12 | 13 | 14 | 15 | 16 | 17 | 18 | 19 | 20 |
| **Nisin cluster** |  |  |  |  |  |  |  |  |  |  |  |  |  |  |  |  |  |  |  |  |
| Transposase for IS element IS904 | CYU10_001648 | + | + | + | + | + | + | + | + | + | + | + | – | + | – | + | – | + | – | + |
| Putative transposase YkgN | CYU10_001649 | + | + | + | + | + | + | + | + | + | + | + | – | + | – | + | – | + | – | + |
| *nisZ/A* | CYU10_001650 | + | + | + | – | – | – | – | + | – | – | – | – | + | + | – | – | – | – | – |
| *nisB* | CYU10_001651 | + | + | + | – | – | – | – | + | – | – | – | – | + | + | – | – | – | – | – |
| *nisT* | CYU10_001652 | + | + | + | – | – | – | – | + | – | – | – | – | + | + | – | – | – | – | – |
| *nisC* | CYU10_001653 | + | + | + | – | – | – | – | + | – | – | – | – | + | + | – | – | – | – | – |
| *nisI* | CYU10_001654 | + | + | + | – | – | – | – | + | – | – | – | – | + | – | – | + | – | + | – |
| *nisP* | CYU10_001655 | + | + | + | – | – | – | – | + | – | – | – | – | + | + | – | – | – | – | – |
| *nisR* | CYU10_001656 | + | + | + | – | – | – | – | + | – | – | – | – | + | + | – | + | – | + | – |
| *nisK* | CYU10_001657 | + | + | + | – | – | – | – | + | – | – | – | – | + | – | – | + | – | + | – |
| *nisF* | CYU10_001658 | + | + | + | + | – | – | – | + | – | – | – | – | + | + | – | + | – | + | – |
| *nisE* | CYU10_001659 | + | + | + | – | – | – | – | + | – | – | – | – | + | + | – | + | – | + | – |
| *nisG* | CYU10_001660 | + | + | + | – | – | – | – | + | – | – | – | – | + | + | – | + | – | + | – |
| Transposase for IS element IS904 | CYU10_001661 | – | – | + | + | – | – | – | – | – | + | – | – | – | – | – | – | + | – | – |
| Transposase | CYU10_001662 | + | + | + | + | + | + | + | – | + | + | + | + | – | – | + | + | + | + | – |
| sacR | CYU10_001663 | + | + | + | – | – | – | – | + | – | – | – | – | + | – | – | + | – | + | – |
| scrB | CYU10_001664 | + | + | + | – | – | – | – | + | – | – | – | – | + | – | – | + | – | + | – |
| scrA | CYU10_001665 | + | + | + | – | – | – | – | + | – | – | – | – | + | + | – | + | – | + | – |
| scrK | CYU10_001666 | + | + | + | – | – | – | – | – | – | – | – | – | – | + | – | + | – | + | – |
| **Colicin V** |  |  |  |  |  |  |  |  |  |  |  |  |  |  |  |  |  |  |  |  |
|  | CYU10_000230 | + | + | + | + | + | + | + | + | + | + | + | + | + | + | + | + | + | + | + |
| **Lysozyme** |  |  |  |  |  |  |  |  |  |  |  |  |  |  |  |  |  |  |  |  |
|  | CYU10_000158 | + | – | – | – | – | – | – | – | – | – | – | – | – | – | – | – | – | – | – |
|  | CYU10_000538 | + | – | – | – | – | – | – | – | – | – | – | – | – | – | – | – | – | – | – |
|  | CYU10_002347 | + | + | – | – | + | – | – | – | – | + | + | – | – | – | – | – | – | + | – |
| +, present; –, absent  1, WFLU12 (highlighted); 2, IO–01; 3, CV56; 4, SO; 5, 184; 6, 229; 7, 275; 8, C10; 9, UC11, 10, UC63; 11, UC77; 12, UL8; 13, UC06; 14, UC08; 15, AI–06; 16, IL1403; 17, KF147; 18, KLDS 40325; 19, NCDO2118; 20, A12 | | | | | | | | | | | | | | | | | | | | |

**Table S9.** Genes of WFLU12 coding for proteins involved in acid stress and bile salt resistance

| **Locus tag ^&^** | **Gene** | **Gene classification** | **EC mumber** | **Product** | **Stress response** | **Reference(s)** |
| --- | --- | --- | --- | --- | --- | --- |
| CYU10_000032 | gadC | Dispensable | – | Glutamate/gamma–aminobutyrate antiporter | Acid stress | Sanders et al., 1999; Lebeer et al., 2008 |
| CYU10_000374 | gpmB | Core | 5.4.2.– | Probable phosphoglycerate mutase GpmB | Acid stress | Even et al., 2003 |
| CYU10_000915 | pgi | Core | 5.3.1.9 | Glucose–6–phosphate isomerase | Acid stress |  |
| CYU10_002142 | tpiA | Core | 5.3.1.1 | Triosephosphate isomerase | Acid stress |  |
| CYU10_001869 | copA | Core | 3.6.3.54 | Probable copper–importing P–type ATPase A | Acid stress/ bile resistance | Summarized by Lebeer et al., 2008 |
| CYU10_001538 | guaA_1 | Core | 6.3.5.2 | Probable GMP synthase [glutamine–hydrolyzing] | Acid stress |  |
| CYU10_002131 | guaA_2 | Core | 6.3.5.2 | Probable GMP synthase [glutamine–hydrolyzing] | Acid stress |  |
| CYU10_002503 (Scaffold 2) | guaA_3 | Core | 6.3.5.2 | GMP synthase [glutamine–hydrolyzing] | Acid stress |  |
| CYU10_002288 | dltD | Core | – | Protein DltD | Acid stress |  |
| CYU10_002291 | dltA | Core | 6.1.1.13 | D–alanine––poly(phosphoribitol) ligase subunit 1 | Acid stress |  |
| CYU10_000781 | dps | Core | 1.16.–.– | DNA protection during starvation protein | Bile resistance |  |
| CYU10_001275 | luxS | Core | 4.4.1.21 | S–ribosylhomocysteine lyase | Bile resistance |  |
| CYU10_001520 | clpE | Core | – | ATP–dependent Clp protease ATP–binding subunit ClpE | Acid stress | Lebeer et al., 2008; Oliveira et al., 2017 |
| CYU10_000089 | ldhA | Core | 1.1.1.27 | L–lactate dehydrogenase 1 | Acid stress | Summarized and searched by Oliveira et al., 2017 |
| CYU10_000090 | pyk | Core | 2.7.1.40 | Pyruvate kinase | Acid stress |  |
| CYU10_000398 | atpC | Core | – | ATP synthase epsilon chain | Acid stress |  |
| CYU10_000399 | atpD | Core | 3.6.3.14 | ATP synthase subunit beta | Acid stress |  |
| CYU10_000400 | atpG | Core | – | ATP synthase gamma chain | Acid stress |  |
| CYU10_000401 | atpA | Core | 3.6.3.14 | ATP synthase subunit alpha | Acid stress |  |
| CYU10_000402 | atpH | Core | – | ATP synthase subunit delta | Acid stress |  |
| CYU10_000403 | atpF | Core | – | ATP synthase subunit b | Acid stress |  |
| CYU10_000404 | atpB | Core | – | ATP synthase subunit a | Acid stress |  |
| CYU10_000405 | atpE | Core | – | ATP synthase subunit c | Acid stress |  |
| CYU10_000997 | gap_1 | Dispensable | 1.2.1.12 | Glyceraldehyde–3–phosphate dehydrogenase | Acid stress |  |
| CYU10_001368 | recA | Core | – | Protein RecA | Acid stress |  |
| CYU10_001376 | yjbM | Core | 2.7.6.5 | GTP pyrophosphokinase YjbM | Acid stress |  |
| CYU10_001404 | groL | Core | – | 60 kDa chaperonin | Acid stress |  |
| CYU10_001418 | sodA | Core | 1.15.1.1 | Superoxide dismutase [Mn] | Acid stress |  |
| CYU10_001522 | gap_2 | Core | 1.2.1.12 | Glyceraldehyde–3–phosphate dehydrogenase | Acid stress |  |
| CYU10_001600 | clpB_1 | Core | – | Chaperone protein ClpB | Acid stress |  |
| CYU10_001914 | ywaC | Core | 2.7.6.5 | GTP pyrophosphokinase YwaC | Acid stress |  |
| CYU10_002466 (Scaffold 2) | clpB_2 | Core | – | Chaperone protein ClpB | Acid stress |  |
| CYU10_001250 | pgk | Core | 2.7.2.3 | Phosphoglycerate kinase | Acid stress/ bile resistance |  |
| CYU10_001285 | eno2 | Core | 4.2.1.11 | Enolase 2 | Acid stress/ bile resistance |  |
| CYU10_001350 | gpmA | Core | 5.4.2.11 | 2,3–bisphosphoglycerate–dependentphosphoglyceratemutase | Acid stress/ bile resistance |  |
| CYU10_001602 | eno | Core | 4.2.1.11 | Enolase | Acid stress/ bile resistance |  |
| CYU10_002021 | dnaK | Core | – | Chaperone protein DnaK | Acid stress/ bile resistance |  |
| CYU10_000438 | pepO | Core | 3.4.24.– | Neutral endopeptidase | Bile resistance |  |
| CYU10_000481 | oppA_1 | Core | – | Oligopeptide–binding protein OppA | Bile resistance |  |
| CYU10_000792 | argS | Core | 6.1.1.19 | Arginine––tRNA ligase | Bile resistance |  |
| CYU10_000829 | rpsE | Core | – | 30S ribosomal protein S5 | Bile resistance |  |
| CYU10_000831 | rplF | Core | – | 50S ribosomal protein L6 | Bile resistance |  |
| CYU10_000835 | rplE | Core | – | 50S ribosomal protein L5 | Bile resistance |  |
| CYU10_000841 | rpsC | Core | – | 30S ribosomal protein S3 | Bile resistance |  |
| CYU10_000846 | rplD | Core | – | 50S ribosomal protein L4 | Bile resistance |  |
| CYU10_001079 | pdhD | Core | 1.8.1.4 | Dihydrolipoyl dehydrogenase | Bile resistance |  |
| CYU10_001357 | oppA_2 | Dispensable | – | Oligopeptide–binding protein OppA | Bile resistance |  |
| CYU10_001358 | oppA_3 | Core | – | Oligopeptide–binding protein OppA | Bile resistance |  |
| CYU10_001453 | pyrG | Core | 6.3.4.2 | CTP synthase | Bile resistance |  |
| CYU10_001484 | oppA_4 | Dispensable | – | Oligopeptide–binding protein OppA | Bile resistance |  |
| CYU10_002417 (Scaffold 2) | nagB | Core | 3.5.99.6 | Glucosamine–6–phosphate deaminase | Bile resistance |  |
| CYU10_000900 | ADH2 | Core | 1.2.1.10 | Acetaldehyde dehydrogenase | Bile resistance | Ruiz et al., 2009 |
| CYU10_000951 | glnA | Dispensable | 6.3.1.2 | Glutamine synthetase | Bile resistance |  |
| CYU10_001396 | pbpA | Core | 3.4.–.– | Penicillin–sensitive transpeptidase | Bile resistance |  |
| CYU10_000552 | clpP_1 | Dispensable | 3.4.21.92 | ATP–dependent Clp protease proteolytic subunit | Acid stress | Sanders et al., 1999 |
| CYU10_001617 | clpP_2 | Core | 3.4.21.92 | ATP–dependent Clp protease proteolytic subunit | Acid stress |  |
| CYU10_000203 | gla_1 | Core | – | Glycerol facilitator–aquaporin gla | Osmotic stress | According to the subsystems features predicted by RAST server (Aziz et al., 2008) |
| CYU10_000803 | gla_2 | Core | – | Glycerol facilitator–aquaporin gla | Osmotic stress |  |
| CYU10_001256 | glpF_1 | Dispensable | – | Glycerol uptake facilitator protein | Osmotic stress |  |
| CYU10_002273 | glpF_2 | Dispensable | – | Glycerol uptake facilitator protein | Osmotic stress |  |
| CYU10_001884 | osmV | Dispensable | 3.6.3.– | Osmoprotectant import ATP–binding protein OsmV | Osmotic stress |  |
| CYU10_002532 (Scaffold 2) | opuAA | Dispensable | 3.6.3.32 | Glycine betaine transport ATP–binding protein OpuAA | Osmotic stress |  |
| CYU10_002533 (Scaffold 2) | gbuB | Core | – | Glycine betaine/carnitine transport permease protein GbuB | Osmotic stress |  |
| CYU10_001885 | opuCB | Core | – | Glycine betaine/carnitine/choline transport system permease protein OpuCB | Osmotic stress |  |
| CYU10_000393 | dedA | Core | – | Protein DedA | Detoxification |  |
|  | | | | | | |

**Table S10.** Genes of WFLU12 coding for bacterial cell surface molecules

| **Locus_tag** | **Gene** | **Gene type^#^** | **Cellular_localization** | **Pfam_product_1** |
| --- | --- | --- | --- | --- |
| CYU10_000037 | – | Singleton | Cellwall | – |
| CYU10_000045 | pgdA_1 | Dispensable | Extracellular | Polysaccharide deacetylase |
| CYU10_000049 | – | Dispensable | Extracellular | Bacterial extracellular solute–binding protein |
| CYU10_000111 | msmE_1 | Dispensable | Extracellular | Bacterial extracellular solute–binding protein |
| CYU10_000163 | – | Dispensable | Extracellular | – |
| CYU10_000197 | – | Dispensable | Cellwall | – |
| CYU10_000293 | – | Dispensable | Extracellular | – |
| CYU10_000339 | – | Dispensable | Extracellular | – |
| CYU10_000481 | oppA_1 | Core | Extracellular | Bacterial extracellular solute–binding proteins |
| CYU10_000542 | – | Dispensable | Extracellular | – |
| CYU10_000544 | yqbO | Dispensable | Extracellular | Phage–related minor tail protein |
| CYU10_000609 | – | Dispensable | Extracellular | Mannosyl–glycoprotein endo–beta–N–acetylglucosaminidase |
| CYU10_000670 | chiD | Dispensable | Extracellular | Glycosyl hydrolases family 18 |
| CYU10_000671 | gbpA | Core | Extracellular | Chitin binding domain |
| CYU10_000731 | – | Dispensable | Extracellular | – |
| CYU10_000901 | – | Dispensable | Extracellular | – |
| CYU10_000920 | DCTPP1 | Dispensable | Extracellular | MazG–like family |
| CYU10_000922 | – | Core | Unknown | Fibronectin–binding protein (FBP) |
| CYU10_000978 | usp45_1 | Dispensable | Extracellular | CHAP domain |
| CYU10_001113 | yxkH | Core | Extracellular | Polysaccharide deacetylase |
| CYU10_001155 | colA | Dispensable | Cellwall | Cna protein B–type domain |
| CYU10_001157 | – | Dispensable | Cellwall | – |
| CYU10_001158 | – | Dispensable | Extracellular | – |
| CYU10_001189 | yunD | Core | Cellwall | Calcineurin–like phosphoesterase |
| CYU10_001294 | pgdA_2 | Core | Extracellular | Polysaccharide deacetylase |
| CYU10_001316 | yfkN | Core | Cellwall | 5'–nucleotidase |
| CYU10_001357 | oppA_2 | Dispensable | Cellwall | Bacterial extracellular solute–binding proteins |
| CYU10_001418 | sodA | Core | Extracellular | Iron/manganese superoxide dismutases |
| CYU10_001484 | oppA_4 | Dispensable | Cellwall | Bacterial extracellular solute–binding proteins |
| CYU10_001486 | EF_0799 | Core | Extracellular | LysM domain |
| CYU10_001626 | icaB | Dispensable | Extracellular | Polysaccharide deacetylase |
| CYU10_001627 | cna | Dispensable | Cellwall | Collagen binding domain |
| CYU10_001635 | – | Singleton | Extracellular | MerR family regulatory protein |
| CYU10_001639 | – | Dispensable | Cellwall | von Willebrand factor type A domain |
| CYU10_001640 | – | Singleton | Cellwall | Cna protein B–type domain |
| CYU10_001641 | – | Singleton | Unknown | Sortase family |
| CYU10_001646 | – | Dispensable | Extracellular | – |
| CYU10_001655 | nisP | Dispensable | Cellwall | Subtilase family |
| CYU10_001676 | ceo_1 | Core | Extracellular | Alanine dehydrogenase/PNT |
| CYU10_001694 | – | Dispensable | Extracellular | – |
| CYU10_001708 | – | Dispensable | Cellwall | – |
| CYU10_001734 | apu | Dispensable | Cellwall | Alpha amylase |
| CYU10_001748 | ypzJ | Core | Extracellular | Nucleic–acid–binding protein containing Zn–ribbon domain (DUF2082) |
| CYU10_001794 | – | Dispensable | Cellwall | – |
| CYU10_001795 | – | Dispensable | Extracellular | – |
| CYU10_001798 | – | Dispensable | Cellwall | – |
| CYU10_001799 | – | Dispensable | Cellwall | – |
| CYU10_001800 | – | Dispensable | Cellwall | Cna protein B–type domain |
| CYU10_001802 | – | Core | Cellwall | – |
| CYU10_001894 | – | Dispensable | Cellwall | – |
| CYU10_001933 | – | Dispensable | Extracellular | – |
| CYU10_001948 | usp45_2 | Dispensable | Extracellular | CHAP domain |
| CYU10_002002 | – | Core | Extracellular | NlpC/P60 family |
| CYU10_002080 | – | Dispensable | Extracellular | – |
| CYU10_002196 | inlJ_1 | Dispensable | Unknown | Internalin |
| CYU10_002197 | inlJ_2 | Dispensable | Cellwall | Internalin |
| CYU10_002200 | SP_0314 | Singleton | Extracellular | Polysaccharide lyase family 8 |
| CYU10_002237 | – | Singleton | Extracellular | Mycoplasma protein of unknown function |
| CYU10_002271 | ceo_3 | Dispensable | Extracellular | – |
| CYU10_002281 | amyS | Dispensable | Extracellular | Alpha amylase |
| CYU10_002302 | – | Dispensable | Cellwall | – |
| CYU10_002305 | – | Dispensable | Extracellular | – |
| CYU10_002347 | CPL9 | Dispensable | Cellwall | Glycosyl hydrolases family 25 |
| CYU10_002357 | – | Dispensable | Cellwall | Procyclic acidic repetitive protein (PARP) |
| CYU10_002370 | divIB | Core | Cellwall | POTRA domain |
| CYU10_002372 | – | Core | Extracellular | – |
| CYU10_002374 | – | Dispensable | Cellwall | – |
| CYU10_002375 | – | Dispensable | Extracellular | – |
| CYU10_002379 | – | Dispensable | Cellwall | – |
| CYU10_002385 | – | Singleton | Extracellular | – |
| CYU10_002410 | – | Dispensable | Extracellular | – |
| ^#^ Genes were identified based on pan-genome analysis using genome sequence of 20 L. lactis strains including the strain WFLU12. The 19 strains include bacteria isolated from dairy product UL8, C10, UC063, 275, 229, Il1403, 184, KLDS 4.0325, and SO, plant, AI-06, KF147, and NCDO 2118; meat, UC08 and UC11; drain water, IO-01; fermented food, A12; and; human, CV56. | | | | |

**Table S11.** Genes of WFLU12 coding for proteins potentially involved in the lipid and vitamin metabolism

| **Locus tag** | **Gene name** | **Gene description** | **Gene type^#^** |
| --- | --- | --- | --- |
| Lipid metabolism |  |  |  |
| CYU10_002419 | atoB_1 | 3-ketoacyl-CoA thiolase (EC 2.3.1.16) @ Acetyl-CoA acetyltransferase (EC 2.3.1.9) | Core |
| CYU10_000418 | atoB_2 | 3-ketoacyl-CoA thiolase (EC 2.3.1.16) @ Acetyl-CoA acetyltransferase (EC 2.3.1.9) | Dispensable |
| Thiamine metabolism |  |  |  |
| CYU10_002294 | thiD | hydroxymethylpyrimidine/phosphomethylpyrimidine kinase | Core |
| CYU10_000504 | iscS | cysteine desulfurase | Core |
| CYU10_001482 | iscS | cysteine desulfurase | Core |
| CYU10_001389 | thiI | tRNA uracil 4-sulfurtransferase | Core |
| CYU10_000257 | dxs | 1-deoxy-D-xylulose-5-phosphate synthase | Dispensable |
| CYU10_002507 | dxs | 1-deoxy-D-xylulose-5-phosphate synthase | Dispensable |
| CYU10_002295 | thiM | hydroxyethylthiazole kinase | Core |
| CYU10_002293 | thiE | thiamine-phosphate pyrophosphorylase | Core |
| CYU10_000641 | rsgA | ribosome biogenesis GTPase / thiamine phosphate phosphatase | Core |
| CYU10_000825 | adk | adenylate kinase | Core |
| CYU10_000414 | tenA | thiaminase (transcriptional activator TenA) | Core |
| Riboflavin metabolism |  |  |  |
| CYU10_002054 | ribBA | 3,4-dihydroxy 2-butanone 4-phosphate synthase / GTP cyclohydrolase II | Core |
| CYU10_002052 | ribD | diaminohydroxyphosphoribosylaminopyrimidine deaminase / 5-amino-6-(5-phosphoribosylamino)uracil reductase | Core |
| CYU10_000055 | ybjI | FMN hydrolase / 5-amino-6-(5-phospho-D-ribitylamino)uracil phosphatase | Dispensable |
| CYU10_000365 | ycsE | 5-amino-6-(5-phospho-D-ribitylamino)uracil phosphatase | Core |
| CYU10_002055 | ribH | 6,7-dimethyl-8-ribityllumazine synthase | Core |
| CYU10_002053 | ribE | riboflavin synthase | Core |
| CYU10_002136 | ribF | riboflavin kinase / FMN adenylyltransferase | Core |
| Vitamin B6 metabolism |  |  |  |
| CYU10_001449 | pdxK | pyridoxine kinase | Core |
| CYU10_001563 | serC | phosphoserine aminotransferase | Core |
| CYU10_000851 | thrC | threonine synthase | Core |
| Nicotinate and nicotinamide metabolism | |  |  |
| CYU10_002111 | pncB | nicotinate phosphoribosyltransferase | Core |
| CYU10_001998 | deoD | purine-nucleoside phosphorylase | Core |
| CYU10_002109 | nadD | nicotinate-nucleotide adenylyltransferase | Core |
| CYU10_002113 | nadE | NAD+ synthase | Dispensable |
| CYU10_001377 | ppnK | NAD+ kinase | Core |
| Pantothenate and CoA biosynthesis | |  |  |
| CYU10_002187 | ilvB | acetolactate synthase I/II/III large subunit | Core |
| CYU10_002259 | ilvB | acetolactate synthase I/II/III large subunit | Core |
| CYU10_002260 | ilvH | acetolactate synthase I/III small subunit | Core |
| CYU10_002261 | ilvC | ketol-acid reductoisomerase | Dispensable |
| CYU10_002258 | ilvD | dihydroxy-acid dehydratase | Core |
| CYU10_000079 | panE | 2-dehydropantoate 2-reductase | Core |
| CYU10_002541 | coaA | type I pantothenate kinase | Core |
| CYU10_001535 | coaB | phosphopantothenate---cysteine ligase (CTP) | Core |
| CYU10_001534 | coaC | phosphopantothenoylcysteine decarboxylase | Core |
| CYU10_000909 | coaD | pantetheine-phosphate adenylyltransferase | Core |
| CYU10_001609 | coaE | dephospho-CoA kinase | Core |
| CYU10_001880 | acpS | holo-[acyl-carrier protein] synthase | Core |
| CYU10_000029 | ilvE | branched-chain amino acid aminotransferase | Core |
| Biotin metabolism |  |  |  |
| CYU10_001817 | fabF | 3-oxoacyl-[acyl-carrier-protein] synthase II | Core |
| CYU10_000421 | fabG | 3-oxoacyl-[acyl-carrier protein] reductase | Core |
| CYU10_001816 | fabG | 3-oxoacyl-[acyl-carrier protein] reductase | Core |
| CYU10_001836 | fabG | 3-oxoacyl-[acyl-carrier protein] reductase | Core |
| CYU10_001528 | fabZ | 3-hydroxyacyl-[acyl-carrier-protein] dehydratase | Dispensable |
| CYU10_001819 | fabZ | 3-hydroxyacyl-[acyl-carrier-protein] dehydratase | Core |
| CYU10_001529 | fabI | enoyl-[acyl-carrier protein] reductase I | Core |
| CYU10_000415 | birA | BirA family transcriptional regulator, biotin operon repressor / biotin---[acetyl-CoA-carboxylase] ligase | Core |
| CYU10_000605 | birA | BirA family transcriptional regulator, biotin operon repressor / biotin---[acetyl-CoA-carboxylase] ligase | Core |
| Lipoic acid metabolism |  |  |  |
| CYU10_001083 | lplA | lipoate---protein ligase | Core |
| Folate biosynthesis |  |  |  |
| CYU10_002165 | GCH1 | GTP cyclohydrolase IA | Dispensable |
| CYU10_002167 | folQ | dihydroneopterin triphosphate pyrophosphohydrolase | Core |
| CYU10_002164 | folB | 7,8-dihydroneopterin aldolase/epimerase/oxygenase | Core |
| CYU10_002166 | folP | dihydropteroate synthase | Core |
| CYU10_002160 | DHFR | dihydrofolate reductase | Core |
| CYU10_002168 | folC | dihydrofolate synthase / folylpolyglutamate synthase | Core |
| CYU10_002054 | ribBA | 3,4-dihydroxy 2-butanone 4-phosphate synthase / GTP cyclohydrolase II | Core |
| CYU10_000071 | pabA | para-aminobenzoate synthetase component II | Core |
| CYU10_000070 | pabBC | para-aminobenzoate synthetase / 4-amino-4-deoxychorismate lyase | Core |
| One carbon pool by folate |  |  |  |
| CYU10_002160 | DHFR | dihydrofolate reductase | Core |
| CYU10_002001 | fhs | formate--tetrahydrofolate ligase | Core |
| CYU10_001895 | folD | methylenetetrahydrofolate dehydrogenase (NADP+) / methenyltetrahydrofolate cyclohydrolase | Core |
| CYU10_001561 | glyA | glycine hydroxymethyltransferase | Core |
| CYU10_002468 | purN | phosphoribosylglycinamide formyltransferase 1 | Core |
| CYU10_002473 | purH | phosphoribosylaminoimidazolecarboxamide formyltransferase / IMP cyclohydrolase | Core |
| CYU10_000594 | MTFMT | methionyl-tRNA formyltransferase | Core |
| CYU10_002451 | thyA | thymidylate synthase | Core |
| CYU10_002284 | metF | methylenetetrahydrofolate reductase (NADPH) | Core |
| CYU10_001179 | MTHFS | 5-formyltetrahydrofolate cyclo-ligase | Core |
| Retinol metabolism |  |  |  |
| CYU10_000443 | adhP | alcohol dehydrogenase, propanol-preferring | Core |
| CYU10_002446 | adh | alcohol dehydrogenase | Core |
| Ascorbate and aldarate metabolism | |  |  |
| CYU10_000208 | ulaC | PTS system, ascorbate-specific IIC component | Dispensable |
| CYU10_000206 | ulaA | PTS system, ascorbate-specific IIA component | Dispensable |
| Porphyrin and chlorophyll metabolism | |  |  |
| CYU10_002423 | hemH | protoporphyrin/coproporphyrin ferrochelatase | Dispensable |
| CYU10_000465 | cobC | alpha-ribazole phosphatase | Dispensable |
| Ubiquinone and other terpenoid-quinone biosynthesis | | |  |
| CYU10_000253 | ubiE | demethylmenaquinone methyltransferase / 2-methoxy-6-polyprenyl-1,4-benzoquinol methylase | Dispensable |
| CYU10_001768 | menF | menaquinone-specific isochorismate synthase | Core |
| CYU10_001767 | menD | 2-succinyl-5-enolpyruvyl-6-hydroxy-3-cyclohexene-1-carboxylate synthase | Core |
| CYU10_001766 | menH | 2-succinyl-6-hydroxy-2,4-cyclohexadiene-1-carboxylate synthase | Dispensable |
| CYU10_001763 | menC | O-succinylbenzoate synthase | Core |
| CYU10_001764 | menE | O-succinylbenzoic acid---CoA ligase | Core |
| CYU10_001765 | menB | naphthoate synthase | Core |
| CYU10_000917 | qorB | NAD(P)H dehydrogenase (quinone) | Dispensable |
| ^#^ Genes were identified based on pan-genome analysis using genome sequence of 20 L. lactis strains including the strain WFLU12. The 19 strains include bacteria isolated from dairy product UL8, C10, UC063, 275, 229, Il1403, 184, KLDS 4.0325, and SO, plant, AI-06, KF147, and NCDO 2118; meat, UC08 and UC11; drain water, IO-01; fermented food, A12; and; human, CV56. | | | |

Reference

1. Shimizu-Kadota, M., Kato, H., Shiwa, Y., Oshima, K., Machii, M., Araya-Kojima, T., Zendo, T., Hattori, M., Sonomoto, K., Yoshikawa, H. Genomic features of *Lactococcus lactis* IO-1, a lactic acid bacterium that utilizes xylose and produces high levels of L-lactic acid. *Biosci. Biotechnol. Biochem.* **2013***.* 77, 1804–1808. 10.1271/bbb.130080.

2. McCulloch, J.A., de Oliveira, V.M., de Almeida Pina, A.V., Pérez-Chaparro, P.J., de Almeida, L.M., de Vasconcelos, J.M., de Oliveira, L.F., da Silva, D.E.A., Rogez, H.L.G., Cretenet, M. Complete genome sequence of *Lactococcus lactis* strain AI06, an endophyte of the Amazonian açaí palm. *Genome Announc.* **2014**, *2(6)*:e01225–01214. 10.1128/genomeA.01225-14.

3. Siezen, R.J., Bayjanov, J., Renckens, B., Wels, M., van Hijum, S.A., Molenaar, D., van Hylckama Vlieg, J.E. Complete genome sequence of *Lactococcus lactis* subsp. *lactis* KF147, a plant-associated lactic acid bacterium. *J Bacteriol*. **2010**, *192*: 2649-2650. 10.1128/JB.00276-10.

4. Oliveira, L.C., Saraiva, T.D., Soares, S.C., Ramos, R.T., Sá, P.H., Carneiro, A.R., Miranda, F., Freire, M., Renan, W., Júnior, A.F. Genome Sequence of *Lactococcus lactis* subsp. *lactis* NCDO 2118, a GABA-Producing Strain*. Genome Announc*. **2014**, *2(5)*:e00980–00914.

5. Kelleher, P.; Bottacini, F.; Mahony, J.; Kilcawley, K.N.; van Sinderen, D. Comparative and functional genomics of the *Lactococcus lactis* taxon; insights into evolution and niche adaptation. *BMC Genomics*. **2017**, *18(1)*, 267. 10.1186/s12864-017-3650-5.

6. Zhao, F.; Ma, H.; Lu, Y.; Teng, K.; Kang, X.; Wang, F.; Yang, X.; Zhong, J. Complete genome sequence of *Lactococcus lactis* S0, an efficient producer of nisin. *J. Biotechnol.* **2015**, *198*, 15–6. 10.1016/j.jbiotec.2015.01.024.

7. Yang, X.; Wang, Y.; Huo, G. Complete Genome Sequence of *Lactococcus lactis* subsp. *lactis* KLDS4.0325. *Genome Announc.* **2013**, *1(6)*. pii: e00962-13. doi: 10.1128/genomeA.00962-13.

8. Bolotin, A., Wincker, P., Mauger, S., Jaillon, O., Malarme, K., Weissenbach, J., Ehrlich, S., Sorokin, A. The complete genome sequence of the lactic acid bacterium *Lactococcus lactis* ssp. *lactis* IL1403. *Genome Res.* **2001**, *11*, 731–753. 10.1101/gr.169701.

9. Gao, Y.; Lu, Y.; Teng, K.L.; Chen, M.L.; Zheng, H.J.; Zhu, Y.Q.; Zhong, J. Complete genome sequence of *Lactococcus lactis*subsp. *lactis* CV56, a probiotic strain isolated from the vaginas of healthy women. *J. Bacteriol.* **2011**, *193*, 2886–2887. 10.1128/JB.00358-11.

10. Kelly, W.J., Altermann, E., Lambie, S.C., Leahy, S.C. Interaction between the genomes of *Lactococcus lactis* and phages of the P335 species. *Front Microbiol.* **2013**, *4*, 257. 10.3389/fmicb.2013.00257.

11. Linares, D.M., Kok, J., Poolman, B. Genome sequences of *Lactococcus lactis* MG1363 (revised) and NZ9000 and comparative physiological studies. *J Bacteriol.* **2010**, *192(21)*, 5806-12. 10.1128/JB.00533-10.

12. Andreevskaya, M., Johansson, P., Laine, P., Smolander, O.P., Sonck, M., Rahkila, R., Jääskeläinen, E., Paulin, L., Auvinen, P., Björkroth, J. Genome sequence and transcriptome analysis of meat-spoilage-associated lactic acid bacterium lactococcus piscium MKFS47. *Appl Environ Microbiol.* **2015**, *81*, 3800–11.

13. Morita, H., Toh, H., Oshima, K., Yoshizaki, M., Kawanishi, M., Nakaya, K., Suzuki, T., Miyauchi, E., Ishii, Y., Tanabe, S., Murakami, M., Hattori, M. Complete genome sequence and comparative analysis of the fish pathogen *Lactococcus* *garvieae*. *PLoS One*, **2011**, *6(8)*,e23184. 10.1371/journal.pone.0023184.
