# Supplementary material for: Genome-Wide Comparison Reveals a Probiotic Strain Lactococcus lactis WFLU12 Isolated from the Gastrointestinal Tract of Olive Flounder (Paralichthys olivaceus) Harboring Genes Supporting Probiotic Action
Source: Mar Drugs. 2018 Apr 24;16(5):140. doi: 10.3390/md16050140 (PMC5983272; doi:10.3390/md16050140)
Supplement: Supplementary file 1 [file marinedrugs-16-00140-s001.zip › marinedrugs-272713 Supplementary information/Supplementary figures (180311).pptx]

## Slide 1
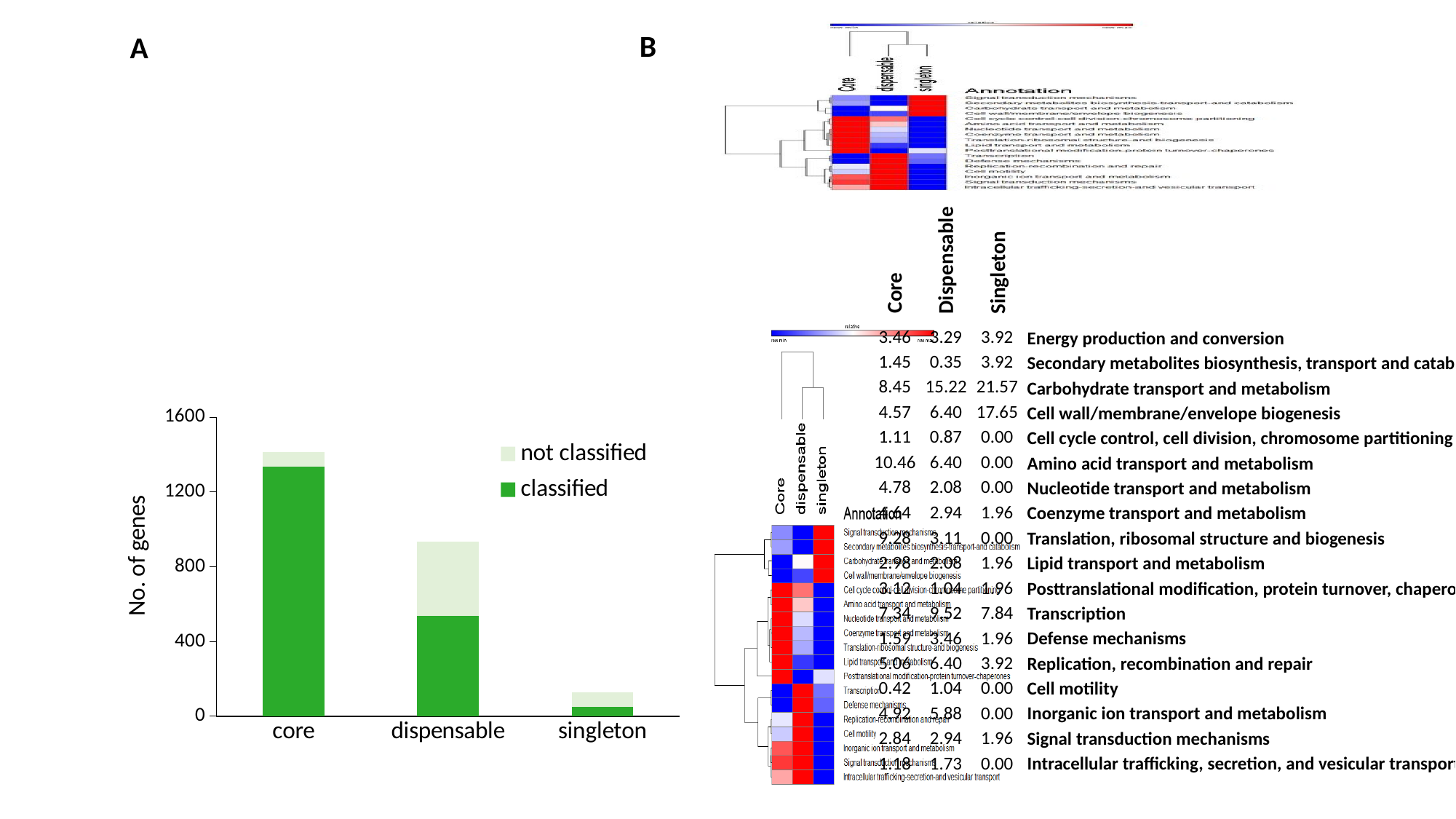

Figure S1. Functional annotation of ortholog groups in the different parts of pan-genome of strain WFLU12. (A) Distribution of ortholog groups, functionally annotated and not annotated using search in the COG database in the different parts of the pan-genome. (B) Scaled heatmap of the distribution of the functional classes among the different parts of pan-genome.
B
A
Dispensable
Core
Singleton
| 3.46 | 3.29 | 3.92 |
| --- | --- | --- |
| 1.45 | 0.35 | 3.92 |
| 8.45 | 15.22 | 21.57 |
| 4.57 | 6.40 | 17.65 |
| 1.11 | 0.87 | 0.00 |
| 10.46 | 6.40 | 0.00 |
| 4.78 | 2.08 | 0.00 |
| 4.64 | 2.94 | 1.96 |
| 9.28 | 3.11 | 0.00 |
| 2.98 | 2.08 | 1.96 |
| 3.12 | 1.04 | 1.96 |
| 7.34 | 9.52 | 7.84 |
| 1.59 | 3.46 | 1.96 |
| 5.06 | 6.40 | 3.92 |
| 0.42 | 1.04 | 0.00 |
| 4.92 | 5.88 | 0.00 |
| 2.84 | 2.94 | 1.96 |
| 1.18 | 1.73 | 0.00 |
| Energy production and conversion |
| --- |
| Secondary metabolites biosynthesis, transport and catabolism |
| Carbohydrate transport and metabolism |
| Cell wall/membrane/envelope biogenesis |
| Cell cycle control, cell division, chromosome partitioning |
| Amino acid transport and metabolism |
| Nucleotide transport and metabolism |
| Coenzyme transport and metabolism |
| Translation, ribosomal structure and biogenesis |
| Lipid transport and metabolism |
| Posttranslational modification, protein turnover, chaperones |
| Transcription |
| Defense mechanisms |
| Replication, recombination and repair |
| Cell motility |
| Inorganic ion transport and metabolism |
| Signal transduction mechanisms |
| Intracellular trafficking, secretion, and vesicular transport |
### Chart
| Category | classified | not classified |
|---|---|---|
| core | 1337.0 | 78.0 |
| dispensable | 536.0 | 400.0 |
| singleton | 49.0 | 80.0 |

## Slide 2
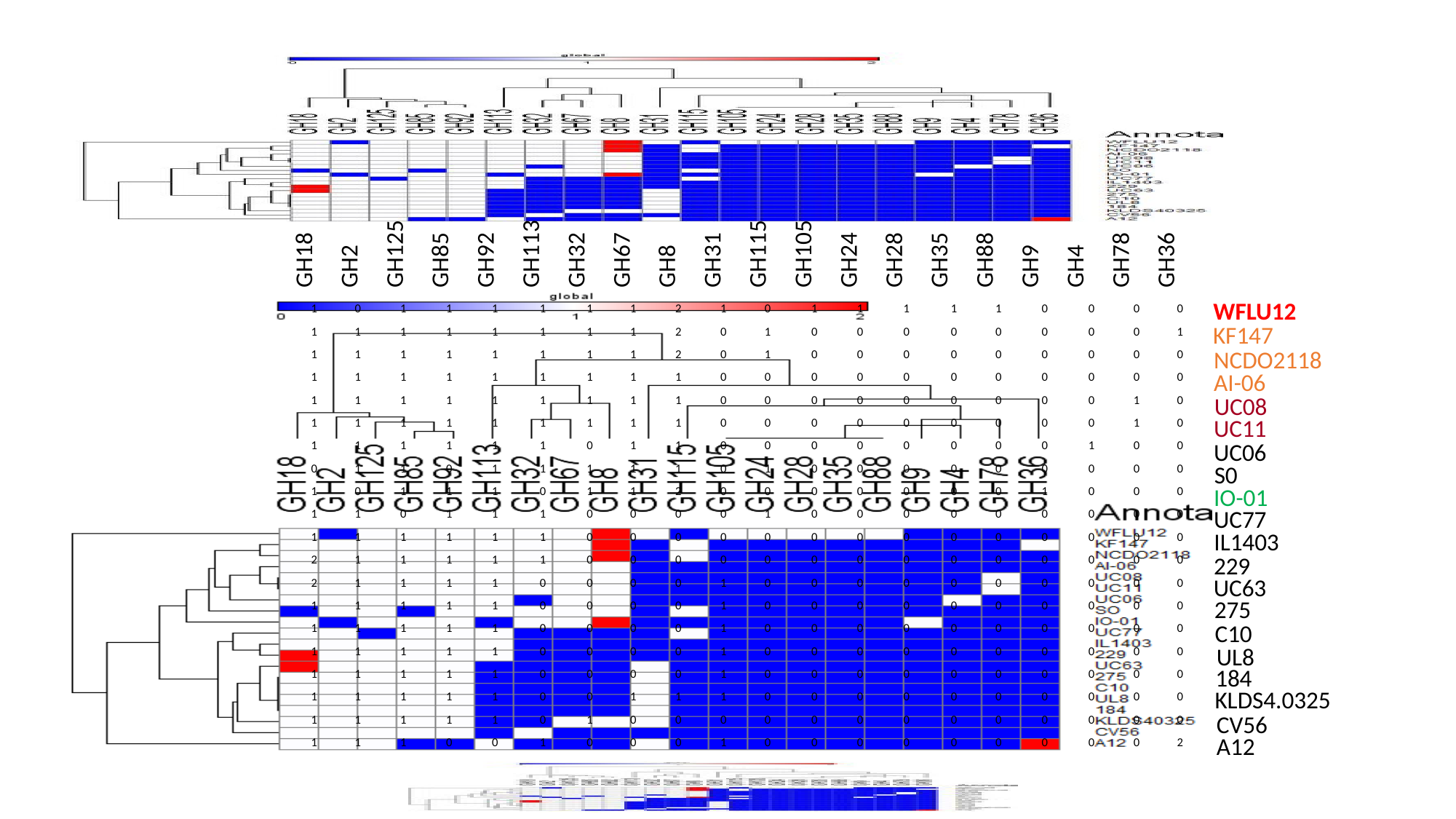

Figure S2. Non-uniform CAZy profiles analysis of L. lactis strains. The numbers of enzyme modules in each genome are shown. Overrepresented (red) and underrepresented modules (blue) are depicted as number of repertoires. Each strain labeled with colour-coded as indicated in Figure 2.
| GH18 | GH2 | GH125 | GH85 | GH92 | GH113 | GH32 | GH67 | GH8 | GH31 | GH115 | GH105 | GH24 | GH28 | GH35 | GH88 | GH9 | GH4 | GH78 | GH36 |
| --- | --- | --- | --- | --- | --- | --- | --- | --- | --- | --- | --- | --- | --- | --- | --- | --- | --- | --- | --- |
WFLU12
| 1 | 0 | 1 | 1 | 1 | 1 | 1 | 1 | 2 | 1 | 0 | 1 | 1 | 1 | 1 | 1 | 0 | 0 | 0 | 0 |
| --- | --- | --- | --- | --- | --- | --- | --- | --- | --- | --- | --- | --- | --- | --- | --- | --- | --- | --- | --- |
| 1 | 1 | 1 | 1 | 1 | 1 | 1 | 1 | 2 | 0 | 1 | 0 | 0 | 0 | 0 | 0 | 0 | 0 | 0 | 1 |
| 1 | 1 | 1 | 1 | 1 | 1 | 1 | 1 | 2 | 0 | 1 | 0 | 0 | 0 | 0 | 0 | 0 | 0 | 0 | 0 |
| 1 | 1 | 1 | 1 | 1 | 1 | 1 | 1 | 1 | 0 | 0 | 0 | 0 | 0 | 0 | 0 | 0 | 0 | 0 | 0 |
| 1 | 1 | 1 | 1 | 1 | 1 | 1 | 1 | 1 | 0 | 0 | 0 | 0 | 0 | 0 | 0 | 0 | 0 | 1 | 0 |
| 1 | 1 | 1 | 1 | 1 | 1 | 1 | 1 | 1 | 0 | 0 | 0 | 0 | 0 | 0 | 0 | 0 | 0 | 1 | 0 |
| 1 | 1 | 1 | 1 | 1 | 1 | 0 | 1 | 1 | 0 | 0 | 0 | 0 | 0 | 0 | 0 | 0 | 1 | 0 | 0 |
| 0 | 1 | 1 | 0 | 1 | 1 | 1 | 1 | 1 | 0 | 1 | 0 | 0 | 0 | 0 | 0 | 0 | 0 | 0 | 0 |
| 1 | 0 | 1 | 1 | 1 | 0 | 1 | 1 | 2 | 0 | 0 | 0 | 0 | 0 | 0 | 0 | 1 | 0 | 0 | 0 |
| 1 | 1 | 0 | 1 | 1 | 1 | 0 | 0 | 0 | 0 | 1 | 0 | 0 | 0 | 0 | 0 | 0 | 0 | 0 | 0 |
| 1 | 1 | 1 | 1 | 1 | 1 | 0 | 0 | 0 | 0 | 0 | 0 | 0 | 0 | 0 | 0 | 0 | 0 | 0 | 0 |
| 2 | 1 | 1 | 1 | 1 | 1 | 0 | 0 | 0 | 0 | 0 | 0 | 0 | 0 | 0 | 0 | 0 | 0 | 0 | 0 |
| 2 | 1 | 1 | 1 | 1 | 0 | 0 | 0 | 0 | 1 | 0 | 0 | 0 | 0 | 0 | 0 | 0 | 0 | 0 | 0 |
| 1 | 1 | 1 | 1 | 1 | 0 | 0 | 0 | 0 | 1 | 0 | 0 | 0 | 0 | 0 | 0 | 0 | 0 | 0 | 0 |
| 1 | 1 | 1 | 1 | 1 | 0 | 0 | 0 | 0 | 1 | 0 | 0 | 0 | 0 | 0 | 0 | 0 | 0 | 0 | 0 |
| 1 | 1 | 1 | 1 | 1 | 0 | 0 | 0 | 0 | 1 | 0 | 0 | 0 | 0 | 0 | 0 | 0 | 0 | 0 | 0 |
| 1 | 1 | 1 | 1 | 1 | 0 | 0 | 0 | 0 | 1 | 0 | 0 | 0 | 0 | 0 | 0 | 0 | 0 | 0 | 0 |
| 1 | 1 | 1 | 1 | 1 | 0 | 0 | 1 | 1 | 1 | 0 | 0 | 0 | 0 | 0 | 0 | 0 | 0 | 0 | 0 |
| 1 | 1 | 1 | 1 | 1 | 0 | 1 | 0 | 0 | 0 | 0 | 0 | 0 | 0 | 0 | 0 | 0 | 0 | 0 | 0 |
| 1 | 1 | 1 | 0 | 0 | 1 | 0 | 0 | 0 | 1 | 0 | 0 | 0 | 0 | 0 | 0 | 0 | 0 | 0 | 2 |
KF147
NCDO2118
AI-06
UC08
UC11
UC06
S0
IO-01
UC77
IL1403
229
UC63
275
C10
UL8
184
KLDS4.0325
CV56
A12
